# Composition of Vaginal Microbiota in Pregnant Women With Aerobic Vaginitis

**DOI:** 10.3389/fcimb.2021.677648

**Published:** 2021-09-09

**Authors:** Kwan Young Oh, Sunghee Lee, Myung-Shin Lee, Myung-Ju Lee, Eunjung Shim, Yun Ha Hwang, Joong Gyu Ha, Yun Seok Yang, In Taek Hwang, Jun Sook Park

**Affiliations:** ^1^Department of Obstetrics and Gynecology, Eulji University School of Medicine, Daejeon, South Korea; ^2^Research Laboratories, Ildong Pharmaceutical Co., Ltd, Hwaseong, South Korea; ^3^Department of Microbiology and Immunology, Eulji University School of Medicine, Daejeon, South Korea

**Keywords:** aerobic vaginitis, vaginal microbiota, dysbiosis, *Lactobacillus*, *Staphylococcus* spp., *Streptococcus* spp., *Enterobacteriaceae*

## Abstract

Vaginal dysbiosis, such as bacterial vaginosis (BV) and aerobic vaginitis (AV), is an important cause of premature birth in pregnant women. However, there is very little research on vaginal microbial distribution in AV compared to that in BV. This study aimed to analyze the composition of the vaginal microbiota of pregnant women with AV using microbial community analysis and identify the causative organism using each criterion of the AV scoring system. Also, we compared the quantification of aerobic bacteria using quantitative polymerase chain reaction (qPCR) and their relative abundances (RA) using metagenomics. This prospective case–control study included 228 pregnant Korean women from our previous study. A wet mount test was conducted on 159 women to diagnose AV using the AV scoring system. Vaginal samples were analyzed using metagenomics, Gram staining for Nugent score determination, conventional culture, and qPCR for *Staphylococcus* spp.*, Streptococcus* spp., and *Enterobacteriaceae*. The relative abundances (RAs) of eleven species showed significant differences among the three groups (Normal flora (NF), mild AV, and moderate AV). Three species including *Lactobacillus crispatus* were significantly lower in the AV groups than in the NF group, while eight species were higher in the AV groups, particularly moderate AV. The decrease in the RA of *L. crispatus* was common in three criteria of the AV scoring system (*Lactobacillary*, WBC, and background flora grades), while it did not show a significant difference among the three grade groups of the toxic leukocyte criterion. Also, the RAs of anaerobes, such as *Gardnerella* and *Megasphaera*, were higher in the AV groups, particularly moderate AV, while the RAs of aerobes were very low (RA < 0.01). Therefore, qPCR was performed for aerobes (*Staphylococcus* spp.*, Streptococcus* spp., and *Enterobacteriaceae*); however, their quantification did not show a higher level in the AV groups when compared to that in the NF group. Therefore, AV might be affected by the RA of *Lactobacillus* spp. and the main anaerobes, such as *Gardnerella* spp. Activation of leukocytes under specific conditions might convert them to toxic leukocytes, despite high levels of *L. crispatus*. Thus, the pathogenesis of AV can be evaluated under such conditions.

## 1 Introduction

Vaginal dysbiosis, such as bacterial vaginosis (BV) and aerobic vaginitis (AV), is a leading cause of premature labor (Hay et al., 1994; Hillier et al., 1995; Goldenberg et al., 2000; Koumans et al., 2007; Goldenberg et al., 2008). Early preterm labor (< 32 weeks of gestation) is thought to be associated with intrauterine infection (Wenstrom et al., 1998; Goldenberg et al., 2008; Gervasi et al., 2012). Therefore, ascending intrauterine infection might be one of the major causes since there have been several studies on the relationship between vaginal infection and premature birth (Wenstrom et al., 1998; Goldenberg et al., 2000; Goldenberg et al., 2008; Gervasi et al., 2012; Oh et al., 2017).

Recently, the development of metagenomics technology has contributed in understanding the community state types of vaginal microorganisms and the microbiota composition in dysbiosis, such as BV, which has been reported to be associated with preterm labor (Ravel et al., 2011; Fox and Eichelberger, 2015). However, the vaginal condition in AV differs from that in BV, and is associated with specific clinical management and distinct clinical risks (Donders et al., 2000; Donders et al., 2009; Donders, 2010). AV is known to 1) decrease the number of *Lactobacillus*, 2) cause severe inflammation, unlike BV, and 3) increase the number of aerobic enteric species, including Group B *Streptococcus (Streptococcus agalactiae)*, *Enterococcus faecalis*, *Escherichia coli*, and *Staphylococcus aureus* (Donders et al., 2000; Donders et al., 2009; Donders, 2010; Donders et al., 2011; Fredricks, 2011; Ghartey et al., 2014; Kaambo et al., 2018). Rumyantseva et al. (2016) attempted to quantify bacteria associated with AV using real-time polymerase chain reaction (qPCR). They reported that there were no significant quantitative differences in the number of *Enterobacteriaceae, Staphylococcus* spp., and *Streptococcus* spp. between the AV group and normal flora (NF). However, they found significant differences in the ratio of the quantification of *Lactobacillus* spp. and aerobic bacteria. Despite its clinical significance, there has been no research on vaginal microbial composition for AV using microbial community analysis, except for a recent study by Wang et al. (2020). The microbial community analysis for AV will increase the understanding of its pathogenesis and will serve as a basis for suggesting guidelines for appropriate treatment.

Therefore, this study aimed to analyze the composition of vaginal microorganisms in AV using metagenomics analysis and identify the main causative bacteria using each element of the AV scoring system. In addition, qPCR and conventional culturing were performed to evaluate the role of *Staphylococcus* spp.*, Streptococcus* spp., and *Enterobacteriaceae*, which are known to cause AV.

## 2 Materials and Methods

### 2.1 Subjects

This study was performed using the same subjects as those in our previous study (Lee et al., 2020), and a prospective case–control study was conducted in 228 Korean pregnant women. As described in our previous study, 16 women were excluded from analysis due to obstetric or medical illness (preeclampsia, five cases; gestational diabetes, seven cases; other medical diseases, four cases). 212 women were selected. However, the wet mount for diagnosis of AV was performed in 159 subjects of them because of the diagnostic limitation of requiring a fresh sample.

Further, vaginal samples were obtained for metagenomic analysis, Gram staining for Nugent score determination, conventional culture, and qPCR for *Staphylococcus* spp.*, Streptococcus* spp., and *Enterobacteriaceae.* This study involving human participants was approved by the Institutional Review Board (IRB) of Eulji University Hospital (IRB No. 2017-07-007-002 and 2020-01-011-002). The patients provided their written informed consent to participate in this study.

### 2.2 Vaginal Sampling

Sampling methods for microbial community analysis, Gram staining, and vaginal pH were described in our previous study (Lee et al., 2020). Samples for wet mounts were obtained from the posterior fornix of the vaginal wall. The cotton swab for the wet mount was inserted in a plain tube containing saline (1 mL) and sent to the laboratory within 10 min, along with the samples for Gram staining and conventional culture. Samples for microbial community and qPCR analyses were stored in a deepfreezer at −80°C until DNA extraction.

### 2.3 Diagnosis of Aerobic Vaginitis

The samples for wet mount were smeared on a slide and were evaluated the status of aerobic vaginitis using phase-contrast microscopy. The two results were compared with each other and reported according to the AV scoring system of Donders (2010) (**Supplementary Table 1**). An AV score of 0–2 was defined as normal flora (NF), 3–4 as mild AV, 5–6 as moderate AV, and above 7 as severe AV.

### 2.4 Conventional Culture

The intravaginal secretions collected for the aerobic culture test were sent to the laboratory using transport media. The cultures were inoculated on sheep blood agar, MacConkey agar, and phenylethyl alcohol (PE) agar plates, and incubated at 37°C for two days. The strains were identified based on culturing, Gram staining, and the Vitek II (bioMerieux, Marcy L’Etoile, France) microbial identification system.

### 2.5 Gram Stain for Nugent Score Determination and Microbial Community Analysis

Gram staining for Nugent score determination and microbial community analysis were performed as described in our previous study (Lee et al., 2020). The Nugent scoring based on Gram staining was used to diagnose the bacterial vaginosis and intermediated flora (Nugent et al., 1991). Nugent scores were obtained for *Lactobacillus*-like, *Mobiluncus*-like, *Gardnerella*-like, and *Bacteroides*-like morphotypes on Gram-stained slides. The subjects were classified into three groups, BV, intermediate flora, and NF, based on the Nugent score.

For microbial community analysis, samples stored at -80°C were defrosted in an icebox (4°C) and vortexed in a FastPrep^®^-24 instrument (MP Biomedicals, USA) by adding 100 μL of samples to the PowerBead Tubes (Garnet 0.79 mm, Qiagen, Helden, Germany) to homogenize each sample. DNA was then extracted from these samples using the QIAamp DNA Mini QIAcube Kit (#51326, Qiagen, Hilden, Germany). DNA quality and quantity were ascertained using Nanodrop apparatus (NanoDrop Technologies, Inc., Wilmington, DE, USA) and stored at -80°C. Following DNA extraction, bacterial genomic DNA was amplified with 16S_V3_341F (5´-TCGTCGGCAGCGTCAGATGTGT ATAAGA GACAGCCTACGGGNGGCWGCAG-3´) and 16S_V4_805R (5´-GTCTCGTGGGCTCGGAGAT GTGTATAAGAGACAGGACTACHVGG GTATCTAATCC-3´) primers, which are specific for V3-V4 hypervariable regions of 16S DNA (Muyzer et al., 1993) with Illumina overhang adaptors. PCR amplification was performed using the following thermal cycling conditions: initial denaturation at 95°C for 3 min, followed by 25 cycles of denaturation at 95°C for 30 s, primer annealing at 55°C for 30 s, and extension at 72°C for 30 s, with a final elongation step at 72°C for 5 min. Secondary amplification for attaching the Illumina Nextera barcode was carried out with the i5 forward and i7 reverse primer (i5: 5´-AATGATACGGCGACCACCGAGATCTACAC-XXXXXXXX-TCGTCGG CAGCGTC-3´; i7: 5´-CAAGCAGAAGACGGCATACGAGAT-XXXXXXXX-GTCTCGTGGGCT CGG-3´; X indicates the barcode region). The secondary amplification conditions were similar to those used for the primary amplification, except that the amplification cycle was set to eight cycles. The PCR product was confirmed using 1.0% agarose gel electrophoresis and visualized under a Gel Doc system (BioRad, Hercules, CA, USA). The amplified products were purified using the CleanPCR kit (CleanNA, Inc., Netherlands) to remove non-target products. The DNA quality and product size were assessed on a Bioanalyzer 2100 (Agilent, Palo Alto, CA, USA) using a DNA 7500 chip. Mixed amplicons were pooled together as equal concentrations, and sequencing was carried out at ChunLab, Inc. (Seoul, Korea) using the Illumina MiSeq Sequencing System (Illumina, USA) manufacturer’s instructions. These processes have been performed the same as our previous study (Lee et al., 2020).

### 2.6 Quantification of Bacteria Using qPCR

qPCR was performed for each bacterial genomic DNA using Taq DNA polymerase (SolGent Co. Ltd., Daejeon, South Korea), and primers/probes for specific bacteria (**Supplementary Table 2**) on a CFX96 Real-Time system C1000 Thermocycler System (Bio-Rad, Hercules, CA). The primers and probes targeting *Enterobacteriaceae, Staphylococcus* spp., and *Streptococcus* spp. were designed to detect the corresponding species/genera as described in a previous study with minor modifications (**Supplementary Table 1**) (Rumyantseva et al., 2016). Template DNA was amplified in a 20-μL PCR mixture with Taq polymerase (SolGent), and the following program was used: 95°C for 2 min, followed by 40 cycles at 95°C for 20 s, 55°C for 40 s, and 72°C for 30 s.

### 2.7 Standard Curve for the Quantification of Staphylococcus spp., Streptococcus spp., and Enterobacteriaceae

*Staphylococcus aureus, Streptococcus vestibularis*, and *Escherichia coli* were selected as representatives of each group for quantification. They were procured from Enzynomics (Daejeon, South Korea), National Culture Collection for Pathogens (NCCP, South Korea), and Korean Collection for Type Cultures (KCTC, South Korea), respectively. For the standard control, *E. coli* and *S. aureus* were cultured in Luria–Bertani (LB) broth (LPS solution, Daejeon, South Korea), and *S. vestibularis* was cultured in tryptic soy broth (MBcell, Seoul, South Korea) at 37°C. After culturing, 100 μL of bacteria was diluted (1:10) in culture media and smeared on agar plates. Further, 100 μL of the cultured bacteria was used for DNA extraction, which was performed using the TaKaRa MiniBEST Bacteria Genomic DNA Extraction Kit (TaKaRa, Tokyo, Japan) according to the manufacturer’s protocol. The extracted genomic DNA was eluted in 100 μL nuclease-free water. A standard curve of DNA/colony-forming unit (CFU) was calculated using comparative analysis of the number of cultured colonies.

### 2.8 Statistical Analysis

The values of relative abundance (RA) were converted into percentages (%) to compare the quantitative differences among the three classified groups and grades in each criterion of the AV scoring system (**Supplementary Table 1**). We analyzed the RA values starting from 0.001% because aerobic bacteria were present at very low levels in the vagina. Statistical significance of the differences among groups was tested using the non-parametric Kruskal–Wallis H test, followed by the Mann–Whitney *U* test, when *p* < 0.05. Bonferroni’s correction test was used to correct multiple comparisons (*p* < 0.05/3). All statistical analyses were performed using SPSS version 18 (SPSS Inc., Chicago, IL, USA).

Linear discriminant analysis (LDA) effect size (LEfSe) was used to explore the potential presence of taxonomic clades that can serve as biomarkers for different classes. Statistically significant groups were reported with LDA scores greater than 3.0. Beta diversity was determined by computing the weighted UniFrac distance matrix. The vaginal microbiota communities between groups were compared using a permutation-based multivariate analysis of variance (PERMANOVA) test with 10,000 replicates and principal coordinate analysis (PCoA).

## 3 Results

### 3.1 Demographic Characteristics of the Subjects

Normal flora was diagnosed in 99 patients, while AV was diagnosed in 60 patients (mild, moderate, and severe AV in 42, 16, and 2 cases, respectively). Since there were only two severe cases, we attempted to analyze the three groups (NF, mild AV, and moderate AV groups) after combining the two severe cases with the moderate cases. The three groups showed no statistically significant differences in their demographic characteristics, such as maternal age, parity, abortion history, rate of preterm birth, delivery weeks, and birth weight (**Table 1**). This might be associated either with the treatment administered to the AV group or the small sample size. Moreover, there were no statistically significant differences in the pH of vaginal discharge and foul odor among the groups. However, the number of women with increased vaginal discharge was higher in the moderate AV group than in the NF group (**Table 1**). The AV group had more positive cases in the conventional culture than in the NF group. In addition, the cases with AV coincided with BV, that is, 9/18 (50.0%) of moderate AV cases overlapped with BV and demonstrated a significant difference, while the cases with candidiasis did not show a significant difference among the three groups.

**Table 1:** The demographic characteristics of pregnant women with the aerobic vaginitis (AV).

	NF	Mild AV	Moderate AV	*p*-value
	(n = 99)	(n = 42)	(n = 18)	
Age (years)	33.90 ± 4.26	34.79 ± 4.25	32.83 ± 3.17	0.112
Parity (No.)	1.14 ± 0.84	1.31 ± 0.78	1.35 ± 0.86	0.292
Abortion (No.)	0.39 ± 0.75	0.45 ± 0.67	0.17 ± 0.73	0.099
Preterm history (No.)	0.32 ± 0.70	0.26 ± 0.49	0.06 ± 0.24	0.278
Gestation age at delivery (weeks)	37.87 ± 3.50	37.55 ± 4.45	39.55 ± 1.35	0.077
Birth Weight at delivery (kg)	3.10 ± 0.56	3.14 ± 0.66	3.28 ± 0.39	0.676
Vaginal pH	4.59 ± 0.65 (60)	4.48 ± 0.40 (33)	4.8 ± 0.61 (17)	0.108
Median (range)	4.5 (4–7)	4.5 (4–5)	5.0 (4–6)	
(test No./total No.)^a^	(60/100)	(33/42)	(17/18)	
Increased vaginal discharge	7/62 (11.3%)	2/33 (6.1%)	4/18 (22.2%)	0.000*
Foul odor	8/54 (12.9%)	4/33 (12.1%)	4/18 (22.2%)	0.561
Positive rate in Conventional culture	3/100 (3.0%)	4/43 (9.3%)	4/18 (22.2%)	0.009*
*Candida albicans* (+) in culture	9/100 (9.0%)	6/43 (14.0%)	3/18 (16.7%)	0.507
Bacterial Vaginosis (N = 16)	2/100 (2.0%)	5/43 (11.6%)	9 (50.0%)	0.000*

NF, normal flora group; Mild AV, mild aerobic vaginitis; Moderate AV, moderate aerobic vaginitis group. The demographic characteristics were compared among the three groups using the non-parametric Kruskal–Wallis test followed by Mann–Whitney U test and Chi-square test (*p-value < 0.05 indicates statistical significance). No. indicates number. The vaginal pH was measured in 110 subjects. a represents the total number of subjects for which pH was obtained/total number of subjects in each group.

### 3.2 Relative Abundances of the Vaginal Samples

**Figure 1:**
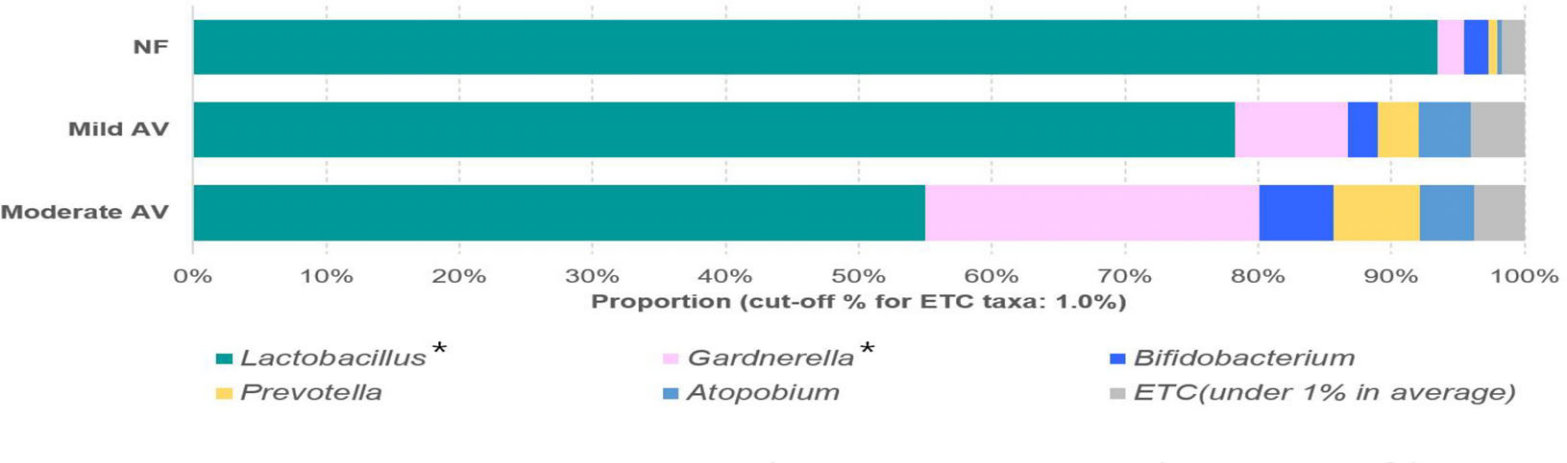
Relative abundances of the three groups, including normal flora, mild aerobic vaginitis, and moderate aerobic vaginitis. Average taxonomic compositions at the genus level among the subjects categorized into the three groups based on the aerobic vaginitis scoring system. The proportion of the bacterial composition was presented at a cut-off of ≥ 1.0%. Asterisk indicates significance between groups (*p < 0.05) as determined using the Kruskal–Wallis test.

Seven operational taxonomic units (OTUs) at the genus level were shown to be significantly different among the three groups (NF, mild AV, and moderate AV) (RA > 0.001%), while only two genera (*Lactobacillus* and *Gardnerella*) showed significant differences (RA > 1%) (**Figure 1**, **Supplementary Table 3**). Eleven OTUs at the species level were significantly different among the three groups (*p* < 0.05) (**Supplementary Table 4**). The RA of *L. crispatus* was lower in the mild and moderate AV groups than in the NF group, furthermore, it was lower in moderate AV than in mild AV. Also, the RA of *L. reuteri* and *Corynebacteriales_CP009312_s*, with very low RA (0.000 ~ 0.002%) was lower in AV groups than NF group. The RA of eight OTUs, (*Gardnerella_ADEP_s*, *Gardnerella*_*ADET_s*, *Megasphaera*; *ADGP_s*, *Dialister*; *KQ960846_s*, *Coriobacteriaceae*; *KQ959671_g*; *KQ959671_s*, *Sneathia sanguinegens*, *Aerococcus christensenii*, *Dialister micraerophilus*) was higher in the mild and moderate AV group than in the NF group. The RA of seven OTUs except that of *Coriobacteriaceae*; *KQ959671_g*; *KQ959671_s* was higher in the moderate AV group than in the NF group (**Supplementary Table 4**).

**Figure 2:**
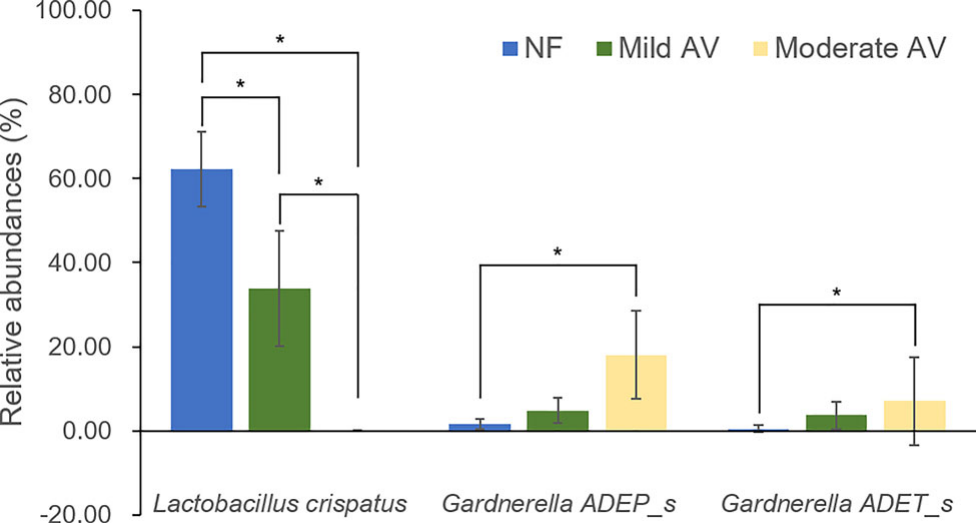
Relative abundances of the three groups, including normal flora, mild aerobic vaginitis, and moderate aerobic vaginitis. Average taxonomic compositions at the species level among the subjects categorized into the three groups based on the aerobic vaginitis scoring system. The proportion of the bacterial composition was presented at a cut-off of ≥ 1.0%. Error bars indicate 95% confidence intervals. Asterisk indicates significance between group (*p < 0.05) as determined using the Kruskal–Wallis test.

Only the RAs of three OTUs (*L. crispatus, G. ADEP_s*, and *G. ADET_s*) showed significant differences (RA > 1%), with *G. ADEP_s* being significantly higher in moderate AV than in mild AV (**Figure 2**, **Supplementary Table 4**).

### 3.3 Diversity Comparison: Species Richness and Diversity Indexes

**Figure 3:**
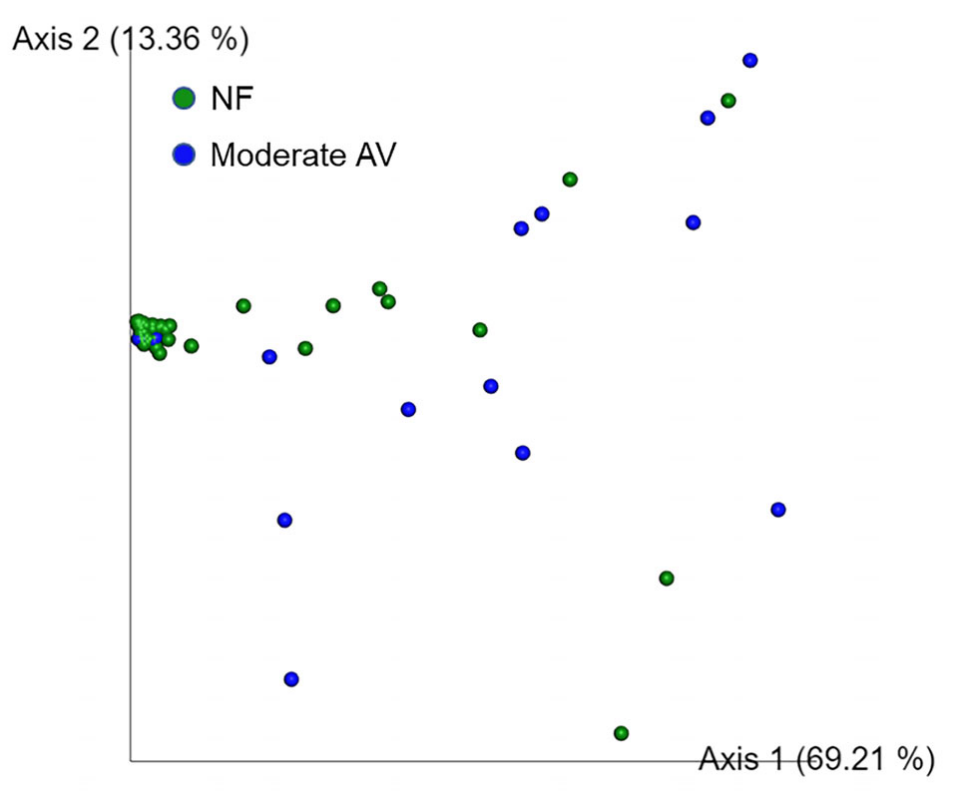
Principal coordinate analysis (PCoA) of the three groups based on weighted UniFrac distances. Each group was classified based on the aerobic vaginitis scoring system. NF and moderate AV represent normal flora (green) and moderate aerobic vaginitis (blue), respectively. Each axis (axis 1 and 2) represents a Principal Component (PC), and the sum of the two axes explanatory power is 82.57%.

The alpha diversity values did not show a statistically significant difference among the three groups. PERMANOVA test revealed significant differences between NF and moderate AV groups as well as between mild and moderate AV groups (*p* = 0.002 and *p* = 0.006, respectively); however, there was no significant difference between the NF and mild AV groups (data not shown). Therefore, we conducted principal coordinate analysis (PCoA) between the NF and moderate AV groups, and found that the NF group was significantly different from the moderate AV group (*p* = 0.002) (**Figure 3**).

**Figure 4:**
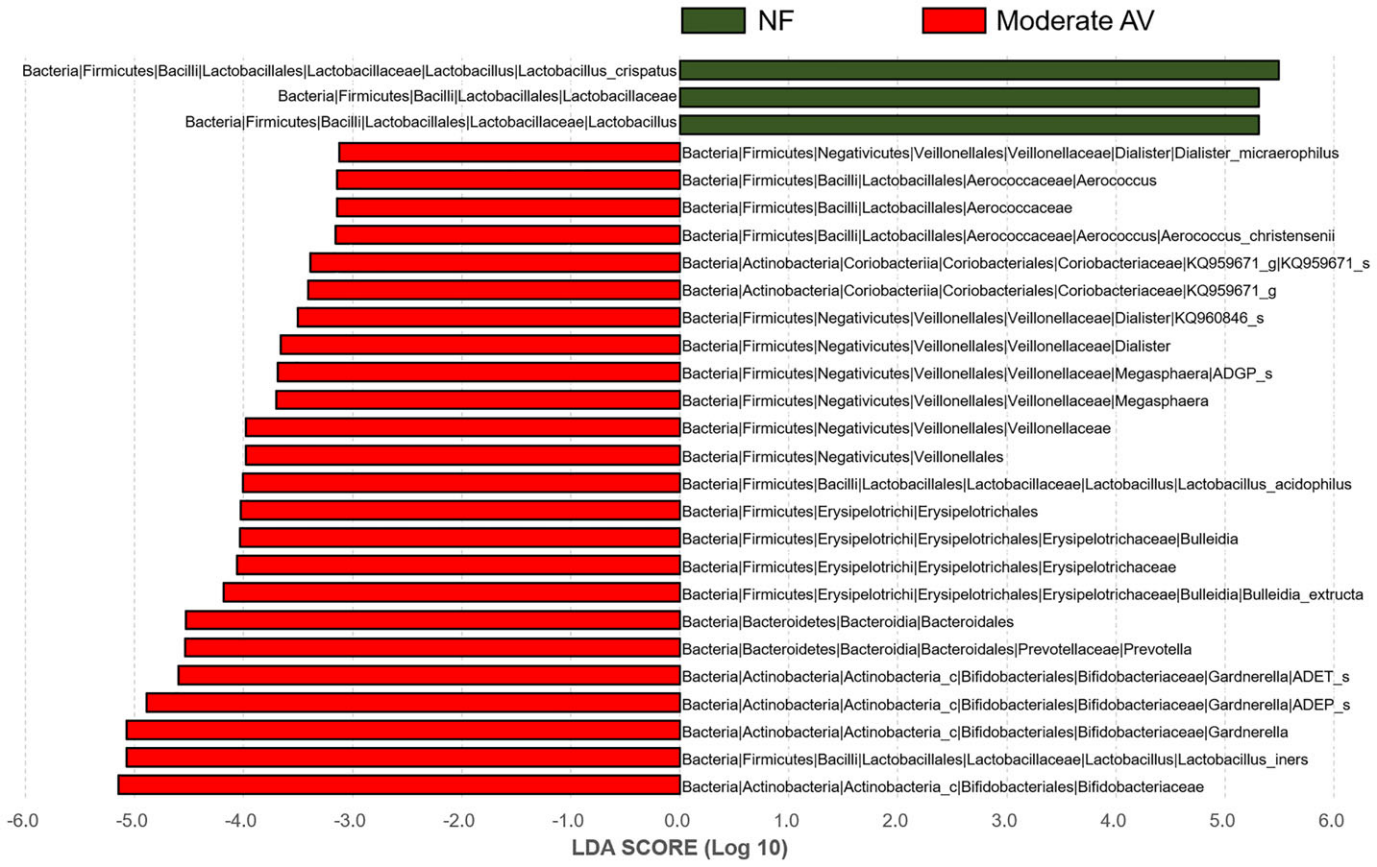
Differential abundance of the bacterial taxa between normal flora and moderate aerobic vaginitis groups. Linear discriminant analysis (LAD) effect size (LEfSe) analysis of the microbial profiles showed significant differences in the abundance of bacterial taxa between normal flora (NF) and moderate aerobic vaginitis (AV) based on their AV scores. NF and moderate AV are indicated by green- and red-colored bars, respectively. The minimum LDA score was set at three.

In addition, we compared and analyzed the LDA score between the NF and moderate AV groups, and developed a cladogram. A cladogram of the moderate AV and NF groups constructed using LEfSe showed that each group was located in different phylogenetic nodes (**Figure 4**). The NF group comprised majorly *Lactobacillus* spp., while the moderate AV group comprised *Gardnerella* spp., *Dialister* spp., and other genera.

### 3.4 Vaginal Microbiota Composition Based on AV Criteria

#### *3.4.1 Lactobacillary* Grade Criterion

There were statistically significant differences in the RA of 35 species among the *Lactobacillary* grade groups (RA > 0.001%). Only *L. crispatus* was lower in grades 1 and 2 than in grade 0 (RA > 1%) (**Supplementary Table 5**). Additionally, there were significant differences between grades 0 and 1 as well as between grades 0 and 2. In patients with RA < 1%, *Bifidobacterium longum* and *Lactobacillus reuteri* were present in significantly higher levels in the grade 0 than in the other groups. They were lower in grade 2 than in grade 0. *L. crispatus* showed a significant difference between grades 0 and 1. However, *B. longum* and *L. reuteri* did not show a significant difference between grades 1 and 2.

The RA of 32 species were higher in the *Lactobacillary* grades 1 or 2 than in grade 0 (RA > 0.001%), while 13 species showed a significant difference (RA > 1.0%) (**Supplementary Table**). Furthermore, 28 OTUs (RAs > 0.001%) showed significant differences between grades 0 and 2, while only 6 OTUs showed significant differences between grades 0 and 1. In addition, four species (*Megasphaera*; *ADGP_s*, *Ruminococcaceae*; *KQ959578_g*; *AY958888_s*, *Dialister*; *KQ960846_s*, and *Dialister micraerophilus*) showed significant differences between grades 1 and 2 (**Supplementary Table 5**).

#### 3.4.2 Toxic Leukocyte Grade Criterion (TL Grade)

In this criterion, the RAs of 17 OTUs were significantly different among the three grade groups (RA > 0.001%) (**Supplementary Table 6**). Only two OTUs *(Atopobium vaginae* and *L. fornicalis)* showed significant differences among the three grade groups, with RA > 1% (*p* = 0.035 and *p* = 0.049, respectively). *A. vaginae* showed a significant difference between grades 0 and 1 (*p* = 0.016), while there were no significant differences between grades 0 and 2 as well as between grades 1 and 2. Moreover, there was no significant difference in the RA of *L. fornicalis* between the grade groups. Furthermore, despite high RA, there was no significant difference in the RA of *L. crispatus, L. iners*, and *Gardnerella*; *ADEP_s* among the three groups. Among the OTUs with RA < 1%, 15 OTUs showed significant differences among the three grade groups. Eleven OTUs were lower in grade 0 than in other grades (black letters), while four OTUs were higher in grade 0 (blue letters) (**Supplementary Table 6**).

#### 3.4.3 White Blood Cell Grade Criterion (WBC Grade)

In this criterion, ten OTUs showed significant differences with RA > 0.001%, while three species (*L. crispatus*, *L. iners*, and *A. vaginae)* showed statistically significant differences with RA > 1% (**Supplementary Table 7**). The RA of *L. crispatus* was lower in grade 2 than in grade 0. In addition, *L. crispatus* showed a significant difference between grades 1 and 2. RA of *L. iners* was higher in grade 2 than in grade 1 (*p* = 0.012), while RA of *A. vaginae* was lower in grade 0 than in the others (*p* = 0.020). In addition, there was a significant difference between grades 0 and 1 (*p* = 0.005) (**Supplementary Table 7**). Despite low levels (RA < 1%), five species showed higher RAs in grade 0 than in the others, (blue letters). In contrast, only *Staphylococcus cohnii* showed higher RA in grade 2 (RA = 0.002) (black letters).

#### 3.4.4 Background Flora Grade Criterion (BF Grade)

In this criterion, the RAs of 13 OTUs showed statistically significant differences with RA > 0.001%, while those of 6 OTUs showed significant differences with RA > 1% (**Supplementary Table 8**). The RA of *L. crispatus* was lower in grades 1 and 2 than in grade 0, while those of other species were higher in grade 2 than grade 0, except *Ralstonia pickettii*, with very low RA (0.000 ~ 0.008%). The RAs of five species (*G. ADEP_s*, *G. ADET_s*, *A. vaginae*, *L. acidophilus*, and *M. ADGP*) were significantly different between grades 1 and 2 and were higher in grade 2 than in grade 0. Overall, none of the cases showed the presence of parabasal epithelial cells among AV scoring system (**Supplementary Table 1**).

**Figure 5:**
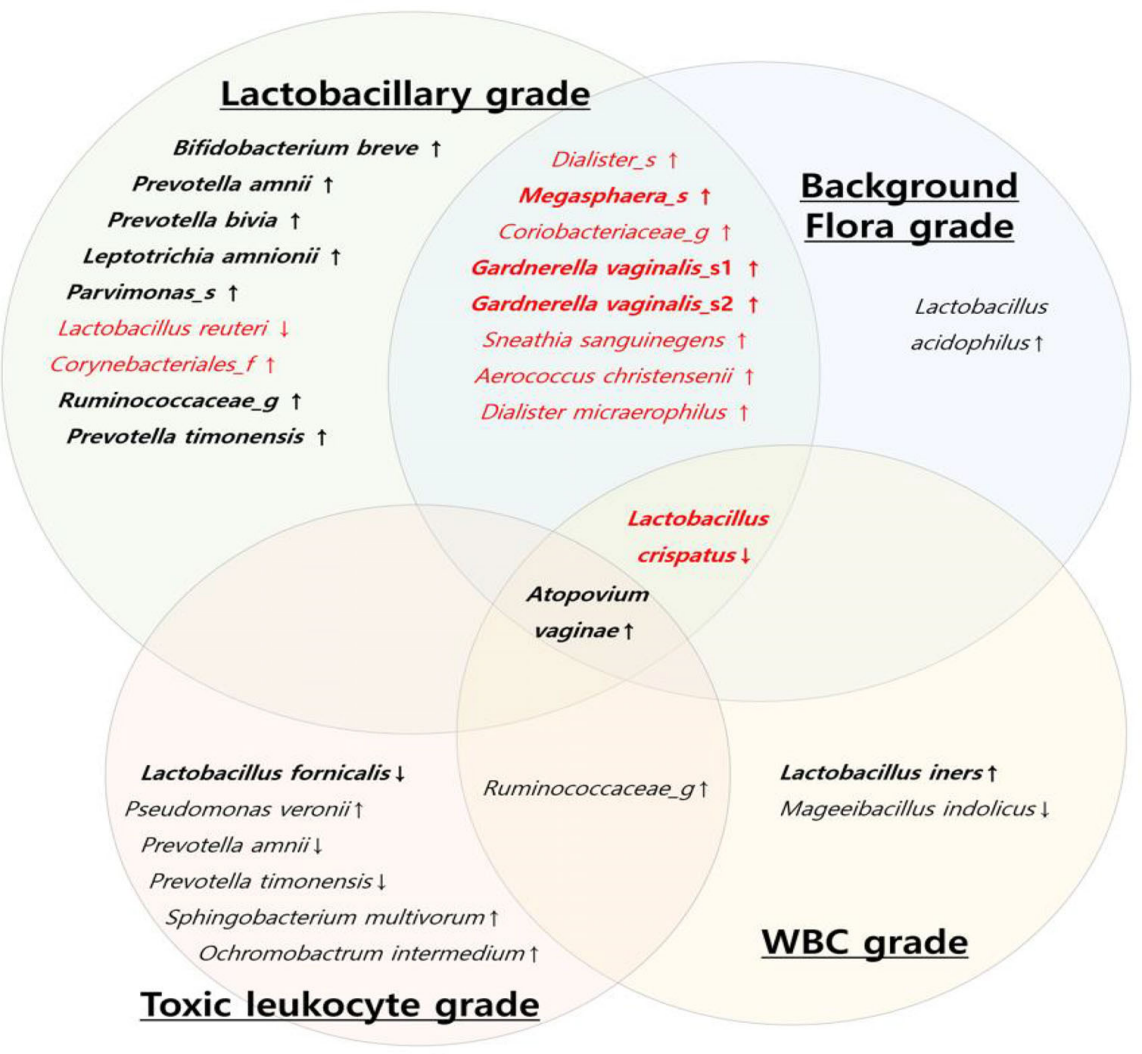
Diagram of the vaginal microbial composition using each criterion of aerobic vaginitis scoring system. (Red letters: vaginal microbiota with a significant difference among the three AV groups (normal flora, mild aerobic vaginitis (AV), and moderate AV), black letters: vaginal microbiota with no significant difference in three AV groups, thick letters: Relative abundance > 1%, thin letters: relative abundance 0.1~ 1%, ↓: significant decrease in AV group, **↑**: significant increase in AV group).

To reveal the microbial characteristics according to each criterion, a diagram was drawn in microorganisms with RAs > 0.1% (**Figure 5**). Based on the above results, decrease in the RA of *L. crispatus* was a common factor in three criteria for the diagnosis of AV, except for the TL grade criterion. Increased RA of *A. vaginae* was a common factor in all the four criteria; however, it did not show a significant difference among the three AV groups (*p* = 0.176) (**Supplementary Table 3**).

In the *Lactobacillary* and BF grade criteria, 10 OTUs showed significant differences among the grades in RAs > 0.1%, as shown in **Figure 5**. The OT Us in the TL and WBC grade criteria revealed different aspects associated with AV (**Figure 5**). Most of the bacteria with significantly higher RAs in the AV groups were anaerobes that were associated with BV; however, the RA of aerobes, such as *Staphylococcus* spp., *Streptococcus* spp., and *Enterobacteriaceae*, was very low (RA < 0.01%).

### 3.5 Quantification of qPCR for Aerobic Bacteria

qPCR for *Staphylococcus* spp.*, Streptococcus* spp., and *Enterobacteriaceae* was performed using the wet mounts of 159 subjects, and seven of them showed high levels of the bacterial DNA in one more strain. Therefore, the qPCR results were analyzed for 152 cases. The amount of DNA from *Staphylococcus* spp. and *Streptococcus* spp. did not show any significant differences among the three groups according to the AV scoring system (**Table 2**). However, the level of DNA from *Enterobacteriaceae* was significantly lower in the mild and moderate AV groups than in the NF group. There was no statistically significant difference between the mild and moderate AV groups. In addition, the sum of the amount of DNA from these bacteria showed a significant difference among the three groups, which was lower in the mild AV group than in the NF group.

**Table 2:** The amount of aerobic bacteria in the vaginal discharge of pregnant women with aerobic vaginitis (AV) using real time PCR (qPCR).

	Normal Flora	Mild AV	Moderate AV	*p-*value
	(n = 96)	(n = 40)	(n = 16)	
*Staphylococcus* spp. (CFU^)	51.05 ± 58.10	31.07 ± 31.98	50.40 ± 43.12^%^	0.062
(range)	(0–315.45)	(0.00–104.35)	(10.74–138.23)	
*Streptococcus* spp. (CFU)	35.91 ± 78.73	22.43 ± 38.71	49.71 ± 78.87	0.239
(range)	(0.00–435.33)	(0.00–189.78)	(0.00–314.95)	
*Enterobacteriaceae* family (CFU)	130.72 ± 79.83	93.25 ± 63.06^$^	67.92 ± 74.97^#^	0.000*
(range)	(48.45–478.90)	(0.00–235.92)	(1.33–290.77)	
Sum (CFU)	217.68 ± 143.58	146.75 ± 62.58^$^	168.02 ± 125.11	0.020*
(range)	(56.98–791.30)	(5.68–295.17)	(37.84–163.83)	

The values of qPCR were compared among the three groups using the non-parametric Kruskal–Wallis test followed by the Mann–Whitney U test using Bonferroni’s correction to adjust the probability (p < 0.05/3). CFU^: colony-forming unit. *p-value < 0.05, indicates statistical significance in the non-parametric Kruskal–Wallis test. ^$^p-value < 0.05/3 between the NF and mild AV groups. ^#^p-value < 0.05/3 between the NF and moderate AV groups. ^%^p-value < 0.05/3 between mild AV and moderate AV groups.

For further evaluation, we analyzed the correlation between the RA of *Staphylococcus* spp.*, Streptococcus* spp., and *Enterobacteriaceae* and the bacterial number using qPCR (**Table 3**). The RA of *Staphylococcus* spp. analyzed using metagenomics was very low (0.00 ~ 0.05%), and there was no correlation with the amount of DNA from *Staphylococcus* spp. analyzed using qPCR. However, the RAs of the *Streptococcus* spp. (0.00 ~ 0.192%) and *Enterobacteriaceae* (0.00 ~ 0.451%) correlated with the quantification of qPCR (*p* = 0.035 and *p* = 0.000, respectively). Four species of the *Streptococcus* genus (*CP006776_s, JYGU_s, S. agalactiae*, and *S. anginosus*) were detected with very low levels of RA using metagenomics. None of the species correlated with the amount of DNA from *Streptococcus* spp. analyzed using qPCR (*p* > 0.05). However, three species of *Enterobacteriaceae* family (*Shigella flexneri*, *Obesumbacterium proteus*, and *Serratia grimesii*) showed a significant correlation with the amount of DNA from *Enterobacteriaceae* family analyzed using qPCR.

**Table 3:** The correlation between the relative abundance (RA) of bacteria using metagenomics and the amount of bacteria using real-time PCR.

	Mean ± SD (range)	Spearman (non-parametric correlation analysis)	*p*-value
***Staphylococcus* spp. RA (%)**	0.003 ± 0.008 (0.00–0.050)	0.029	0.645
* Staphylococcus argenteus* (%)	0.000+0.001 (0.00–0.007)	−0.014	0.837
* Staphylococcus cohnii* (%)	0.001 ± 0.004 (0.00–0.040)	0.025	0.697
* Staphylococcus epidermidis* (%)	0.001 ± 0.005 (0.00–0.043)	0.017	0.795
* Staphylococcus hominis* (%)	0.001 ± 0.004 (0.00–0.037)	0.020	0.767
*** Staphylococcus* spp. PCR (CFU)**	**45.72 ± 51.47 (0.00–315.45)**		
***Streptococcus* spp. RA** (%)	0.003 ± 0.017 (0.00–0.192)	0.135	0.035*
* CP006776_s* (%)	0.001 ± 0.003 (0.00–0.028)	0.085	0.203
* JYGU_s* (%)	0.001 *±* 0.003 (0.00–0.025)	0.096	0.149
* Streptococcus agalactiae* (%)	0.000 ± 0.003 (0.00–0.034)	0.093	0.164
* Streptococcus anginosus* (%)	0.003 ± 0.016 (0.00–0.192)	0.093	0.164
*** Streptococcus* spp. PCR (CFU)**	**33.82 ± 70.49 (0.00–435.33)**		
***Enterobacteriaceae* RA** (%)	0.006 ± 0.043 (0.00–0.451)	0.303	0.000*
* Escherichia*; *Shigella flexneri* (%)	0.001 ± 0.007 (0.00–0.082)	0.195	0.003*
* Hafnia*; *Obesumbacterium proteus* (%)	0.001 ± 0.011 (0.00–0.125)	0.184	0.006*
* Serratia*; *Serratia grimesii* (%)	0.001 ± 0.011 (0.00–0.125)	0.181	0.006*
*** Enterobacteriaceae PCR* (CFU)**	**114.25 ± 78.18 (0.00–478.90)**		
**PCR Sum (CFU)**	**193.78 ± 128.67 (5.68–791.30)**		

Each qPCR values and RA from metagenomics were analyzed using Spearman test (non-parametric correlation analysis) (p < 0.05).

### 3.6 Comparison of Vaginal Microbiota Composition Between Two Types of Moderate AV

There were 18 cases with moderate AV, of which nine cases were coincidental with BV (**Table 1**). Therefore, we classified them into two types [moderate AV-1 (moderate AV without BV) and moderate AV-2 (moderate AV with BV)] to compare the vaginal microbiota composition (**Supplementary Table 9**). The RAs of six OTUs showed significant differences between moderate AV-1 and AV-2. Four species (*G. vaginalis_ADET_s, Atopobium vaginae, Megasphera_ADGP_s*, and *Dialister KQ960846_s*) showed significant differences with RA > 1%. Three species (*G. vaginalis_s2, M. ADGP_s*, and *Dialister KQ960846*) showed higher RAs in moderate AV-2 than in moderate AV-1, whereas only *A. vaginae* had lower RA in moderate AV-2 than in moderate AV-1. Two species (*Aerococcus christensenii* and *Dialister micraerophilus*) showed significant differences between the two types (RA < 1%).

*L. iners and G. vaginalis_s1* (*Gardnerella*; *ADEP_s*) showed higher RA in both groups; however, there were no significant differences (**Supplementary Table 9**). *Prevotella bivia* and *Prevotella buccalis* also showed higher RA in moderate AV-2 than in moderate AV-1, with no significant difference. *L. crispatus* showed a very low level without significant differences between the two groups.

**Figure 6:**
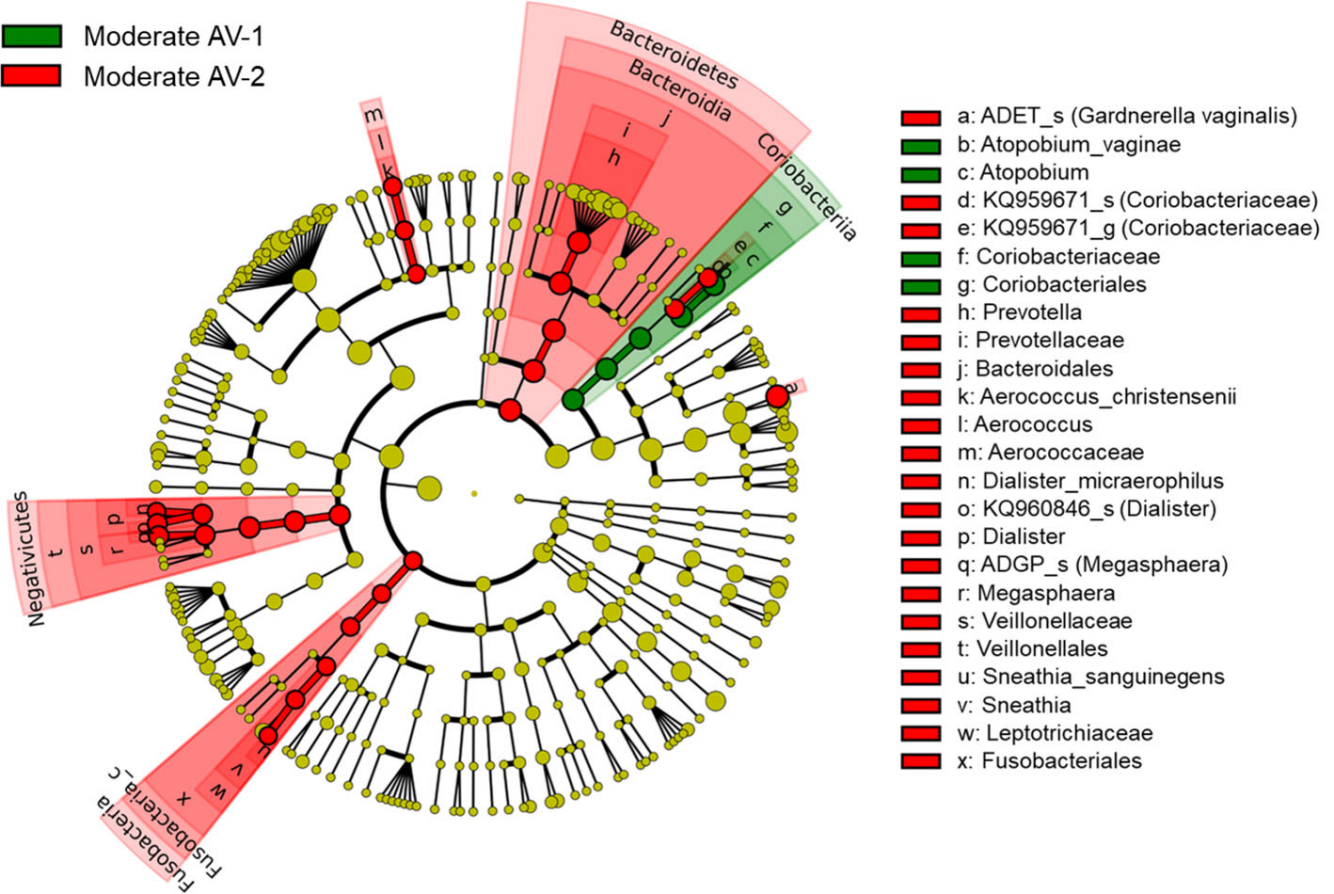
The cladogram based on linear discriminant analysis effect size (LEfSe) of the moderate AV-1 (moderate AV without BV) and the moderate AV-2 (moderate AV with BV). Colors on the cladogram indicate the following: green (moderate AV-1) and red (moderate AV-2). Significantly abundant bacterial groups identified in this study are shown in the list on the right side.

In addition, we compared and analyzed the LDA score between the two types of moderate AV groups and developed a cladogram. A cladogram of the moderate AV-1 and AV-2 groups constructed using LEfSe showed that each group was located in different phylogenetic nodes (**Figure 6**). The moderate AV-1 group included *Coriobacteriales*; *Coriobacteriaceae*; *Atopobium*; *Atopobium vaginae*, while moderate AV-2 included *Gardnerella_ADET_s*, *Dialister* spp., *Megasphaera* spp., and other genera.

## 4 Discussion

In this study, we analyzed the vaginal microbiota composition in women with AV using each criterion of the AV scoring system to evaluate its role in the disease. In this study, we reveal three aspects as follows: 1) The RA of *L. crispatus* in the AV group was lower than that in the NF group, and RAs of anaerobes, such as *Gardnerella* spp., were detected at higher levels in the AV group. In particular, the RA of *L. crispatus* showed significant differences among the three grade groups in three of the four criteria for AV diagnosis. However, in the TL grade criterion, the RA of *L. crispatus* did not show a significant difference among the three grades. 2) The RA of aerobes, such as *Staphylococcus* spp. and *Streptococcus* spp., were low, and they did not show any significant increase in the AV group. 3) Moderate AV was classified into two types: AV-1 type (moderate AV without BV) and AV-2 type (moderate AV with BV), and they revealed different vaginal microbiota compositions.

The first finding in our study reveals that three of the four criteria for AV diagnosis were associated with a decrease in the RA of *L. crispatus.* Increased RA of *L. crispatus* may play an important role in preventing AV because it shows a common intersection in three criteria of the AV scoring system. It is known that the vaginal microbial environment rich in *L. crispatus* indicates good vaginal health because of its anti-bacterial effect (Nugent et al., 1991; Sun et al., 2017; Kaambo et al., 2018; Marziali et al., 2019; Cohen et al., 2020). Therefore, the *Lactobacillary* grade has been included in the diagnostic criteria for both AV and BV (Nugent et al., 1991; Donders, 2010). The Nugent scoring system, which is known as the diagnostic criterion for BV, contains an abundance of *Lactobacillus* spp. Therefore, the two diseases have a predisposition to overlap. In addition, the AV group showed an increase in the RA of anaerobes, such as *Gardnerella* spp. These conditions were similar to those for BV. Therefore, we used the Nugent scoring system and detected BV along with moderate AV in approximately 50% of the women. This could be due to an overlap of the BF and *Lactobacillary* grade criteria of the AV scoring system with the elements of the Nugent scoring system. Ten OTUs at the species level with RA > 0.1% were found in both the criteria. In addition to the decrease in *L. crispatus*, nine species of anaerobes were found to increase with significant differences, indicating BV.

However, the criteria for TL and WBC grades showed slightly different findings. In the TL grade criterion, the RA of *L. crispatus* did not show significant differences among the three grades. In addition, there were no significant differences in the RA of *G. vaginalis* and *Prevotella*. Only two species showed significant differences among the three grade groups for RA > 1%. In the WBC grade criterion, *L. crispatus* had higher RA in grade 0 than in other grades, while *L. iners* and *A. vaginae* had significantly lower RA in grade 0 than in other grades. Six OTUs with low RA (< 1%) showed higher RA in grade 0 than in the others. Only *Staphylococcus cohnii* showed a higher RA (0.002%) in grade 2 than in the others (*p* = 0.018). The presence of parabasal cells indicates high inflammation; however, this was not observed in our study.

Although AV and BV are types of vaginal dysbiosis, no inflammation has been reported in BV (Muzny et al., 2019), whereas characteristic inflammation has been reported in AV (Donders et al., 2000; Donders et al., 2009; Donders, 2010). Activation of immune function might increase the number of WBCs and toxic leukocytes, which are not found in BV. This aspect is presumed to play an important role in elucidating the pathophysiology of AVs. Many researchers have reported that certain aerobic strains can express lipopolysaccharide or stimulate the innate immunity to form toxic leukocytes (Fidel et al., 1994; Elovitz et al., 2003; Ilievski et al., 2007; Thaxton et al., 2009; Koga et al., 2009; Cappelletti et al., 2020). However, our study demonstrated that there were no significant differences in RAs of *Staphylococcus* spp.*, Streptococcus* spp., and *Enterobacteriaceae* among the three AV groups, which contradicts the results from the study of Wang et al. (2020).

AV depends on the presence of *L. crispatus* and anaerobes with higher RAs than the action of individual aerobic strains. Therefore, it may be difficult to diagnose AV using individual aerobic strains. Therefore, the detection of inflammatory markers, such as secreted proteins and short-chained fatty acids, can be developed as a potential diagnostic method (Chen et al., 1979; Briselden et al., 1992; Cauci and Culhane, 2011; Nelson et al., 2015). However, detection of the presence of several known pathogens, such as *S. agalactiae*, using qPCR is a widely used diagnostic tool. In addition, it also facilitates the evaluation of the pathophysiology of AV because this disease is caused by abnormal vaginal microbiota. Most of the aerobes originating from the intestine (Donders et al., 2000; Donders et al., 2009; Donders, 2010; Donders et al., 2011; Fredricks, 2011; Petricevic et al., 2013; Ghartey et al., 2014; Kaambo et al., 2018; Martín et al., 2019; Nugeyre et al., 2019), which are known to cause AV, were observed in metagenomics in a very small amount (RA < 0.01%), making statistical processing difficult.

Therefore, we performed qPCR for aerobes, such as *Staphylococcus* spp., *Streptococcus* spp., and *Enterobacteriaceae*. However, their quantification did not show any significant increase in the AV groups. In contrast, the amount of *Enterobacteriaceae* decreased in the AV group compared to that in the NF group. Therefore, we analyzed strains with an RA value of 0.001% or higher using metagenomic analysis, and compared the values with the quantification of qPCR for *Staphylococcus* spp., *Streptococcus* spp., and *Enterobacteriaceae*. As a result, four strains were identified in *Staphylococcus* spp. and *Streptococcus* spp., and three species in *Enterobacteriaceae*. In addition, to prevent the loss of strains with fewer RAs, *Staphylococcus* spp. and *Streptococcus* spp. were analyzed at the genus level and *Enterobacteriaceae* at the family level to compare the values with that obtained from quantification of qPCR. In metagenomics, the RA of the *Staphylococcus* genus was very low (0.00–0.05%) and it did not correlate with the quantification of qPCR for *Staphylococcus* spp., while those of *Streptococcus* spp. and *Enterobacteriaceae* correlated with the quantification of qPCR (*p* = 0.035 and *p* = 0.00, respectively). The RAs of each species of the *Streptococcus* genus did not correlate with the quantification of qPCR for *Streptococcus* spp. (*p* > 0.05), and this could be associated with very low RA. However, three species of *Enterobacteriaceae* family (*S. flexneri, O. proteus*, and *S. grimesii)* significantly correlated with the quantification of qPCR for *Enterobacteriaceae* (*p* < 0.05). These microorganisms may be commensals or normal flora, and therefore, they were presumed to be unable to induce inflammation, as reported for AV.

These results can be interpreted in two ways. 1) It may be important to determine whether the microbiota themselves can act as pathogens. 2) Since the vaginal environment is anaerobic and facilitates the growth of anaerobes, AV might be affected by anaerobic conditions. However, it is difficult to elucidate in the pathophysiology of AV because only few anaerobic pathogens are known. In addition, very little information is available on the infectivity of certain strains classified as pathogens. However, it has been reported that they have different pathological characteristics according to their genotypes (Alves et al., 2014; Vaneechoutte et al., 2019). Therefore, further studies are required to classify these pathological characteristics. Furthermore, the pathogenicity can be determined depending on the composition of the vaginal ecosystem. A vaginal ecosystem with high RA of *L. crispatus* might suppress pathogens. However, Muzny et al. (2019) reported that a biofilm formed by *G. vaginalis* (GV) indicates destruction of the vaginal mucosal layer. This enables pathogens to easily bind to the Toll-like receptors (TLR) of the vaginal epithelium and secrete chemokines, leading to inflammation. In addition, Libby et al. (2008) reported that *A. vaginae* induces innate immunity in an *in vitro* model. There is a possibility that *A. vaginae* can induce inflammation in BV, or in the case of AV, it is assumed that certain pathogens, such as *Staphylococcus* or *Streptococcus*, can cause vaginal infection even in small numbers. In our study, there was no statistically significant difference in the RA of *A. vaginae* between the AV and NF groups (*p* = 0.176); however, all four criteria of the AV scoring system reported significant differences among the grade groups. Further studies using *in vitro* or *in vivo* experiments are required in the future.

The second finding revealed low levels of *Staphylococcus* spp. and *Streptococcus* spp., when detected using qPCR, and they did not show any significant increase in the AV group. Therefore, their clinical manifestations may depend on the vaginal ecosystem surrounding them. This result corroborates the results reported by Rumyantseva et al. (2016). However, Wang et al. (2020) reported higher RA of aerobes in the AV group than in the NF group. This discrepancy may be due to differences in the subjects, such as pregnant state, race, and inhabitance. Further investigation is required to eliminate these discrepancies.

The third finding of our study reveals that moderate AV consisted of two types (AV-1 and AV-2) depending on the presence of BV. The microbiota composition common to the two types was low RA of *L. crispatus* and high RAs of *L. iners* and *G. vaginalis_s1*. Three species (*G. vaginalis_s2, Megasphaera ADGP_s*, and *Dialister KQ960846*) showed higher RAs in moderate AV-2 than in moderate AV-1, whereas only *A. vaginae* had lower RA in moderate AV-2 than moderate AV-1. In addition, *Prevotella bivia* and *P. amnii* showed higher RA in moderate AV-2 than in AV-1, although the difference was not statistically significant.

Some studies have reported that *G. vaginalis* and *P. bivia* have a symbiotic relationship that facilitates BV development (Pybus and Onderdonk, 1997; Muzny et al., 2019). Therefore, a similar mechanism may regulate moderate AV-2. In moderate AV, the major bacteria were *L. iners* and *G. vaginalis* (RA = 65.6% and RA = 15.8%, respectively), and RA of *L. crispatus* was decreased in moderate AV. This may decrease the immunity of the host. Under these conditions, certain pathogens may attack the host, thereby causing AV. Additionally, in the presence of *G. vaginalis* and *P. bivia*, coordination and proliferation of other anaerobes may lead to the development of AV-2. Under these conditions, several pathogens or opportunistic microorganisms attack the host.

In moderate AV-1 type, certain pathogens or opportunistic strains may induce inflammation by activating the innate immunity under suppressed RA of *L. crispatus* and increased RAs of *G. vaginalis* and *A. vaginae* (Krohn et al., 1991; Ferris et al., 2004; Bradshaw et al., 2006; Hardy et al., 2016). *A. vaginae* could be considered as the causative agent for moderate AV-1 due its higher RA, as mentioned by Libby et al. (2008). Moreover, it also depends on the ability of pathogens or opportunistic microorganisms to induce inflammation. However, little information is available and further studies are required to understand the pathophysiology of AV.

In summary, we analyzed the vaginal microbiota composition of pregnant women with AV. Our study provides important data for evaluating AV pathophysiology in the future.

## Data Availability Statement

The datasets generated for this article can be found in European Nucleotide Archive (ENA) using the accession number PRJEB34614 (https://www.ebi.ac.uk/ena/browser/view/PRJEB34614).

## Ethics Statement

This study involving human participants was approved by the Institutional Review Board (IRB) of Eulji University Hospital (IRB No. 2017-07-007-002 and 2020-01-011-002). The patients/participants provided their written informed consent to participate in this study.

## Author Contributions

KO and SL conceived and designed the study. ES, YH, JH, YY, IH, and JP helped in data acquisition. KO, SL, ES, M-SL, and M-JL analyzed and interpreted the data. KO, SL, M-SL, and M-JL contributed to the writing of the manuscript. All authors contributed to the article and approved the submitted version.

## Funding

This work was supported by EMBRI Grants 2020 EMBRI-DJ 0002 from Eulji University and The National Research Foundation of Korea Grant funded by the Korean Government (NFR- 2017R1A2B1007810).

## Conflict of Interest

SL was employed by ILDONG Pharmaceutical Co. Ltd.

The remaining authors declare that the research was conducted in the absence of any commercial or financial relationships that could be construed as a potential conflict of interest.

## Publisher’s Note

All claims expressed in this article are solely those of the authors and do not necessarily represent those of their affiliated organizations, or those of the publisher, the editors and the reviewers. Any product that may be evaluated in this article, or claim that may be made by its manufacturer, is not guaranteed or endorsed by the publisher.

## Abbreviations

AV, aerobic vaginitis; BV, bacterial vaginosis; NF, normal flora; qPCR, real time polymerase chain reaction; RA, relative abundance; OTUs, operative taxonomic units; PCoA, principal coordinate analysis.
