# Supplementary material for: Composition of Vaginal Microbiota in Pregnant Women With Aerobic Vaginitis
Source: Front Cell Infect Microbiol. 2021 Sep 9;11:677648. doi: 10.3389/fcimb.2021.677648 (PMC8458944; doi:10.3389/fcimb.2021.677648)
Supplement: Supplementary file 1 [file Table_1.docx]

Supplementary Material

# Supplementary Table 1. Criteria for the microscopic diagnosis of Aerobic Vaginitis (AV).

| **AV score** | **Lactobacillary grades (LBG)** | **Number of leukocytes** | **Proportion of toxic leukocytes*** | **Background flora** | **Proportion of parabasal epitheliocytes (PBC)** |
| --- | --- | --- | --- | --- | --- |
| 0 | I and IIa | < 10/hpf | None or sporadic | Unremarkable  or cytolysis | None or <1% |
| 1 | IIb | >10/hpf and  < 10/epithelial cell | <50% of leukocytes | Small bacilli | < 10% |
| 2 | III | >10/epithelial cell | >50% of leukocytes | Cocci or chains | >10% |

# ( x 400 magnification, phase contrast microscope).

*Toxic leukocytes ; leukocytes that are round and bloated, containing swirling lysosomes.

# Supplementary Table 2. Primers and probes used for amplification for aerobic bacteria.

| Bacteria | Primer, probe | Concentration (pmol/react ) |
| --- | --- | --- |
| *Enterobacteriacae* | gCg-gCC-CCC-Tgg-ACg-AAg-A | 0.7 |
| *Enterobacteriacae* | gCC-TCA-Agg-gCA-CAA-CCT-CCA-A | 0.7 |
| *Enterobacteriacae* | Cgg-TTC-AAg-ACC-ACA-ACC-TCT-AA | 0.7 |
| *Enterobacteriacae* | (FAM)CgC-TCA-ggT-gCg-AAA-gCg-Tgg-g(BHQ1) | 0.5 |
| *Staphylococcus spp.* | gCT-ACA-CAC-gTg-CTA-CAA-Tgg-ACA-A | 0.7 |
| *Staphylococcus spp.* | CgT-ATT-CAC-CgT-AgC-ATg-CTg-ATC-TA | 0.7 |
| *Staphylococcus spp.* | (HEX)CAg-CgA-AAC-CgC-gAg-gTC-AAg-C(RTQ) | 0.5 |
| *Staphylococcus spp.* | (HEX)-CTA-AAC-CgC-gAg-gTC-ATg-C(RTQ) | 0.5 |
| *Streptococcus spp* | gCT-ACA-CAC-gTg-CTA-CAA-Tgg-TT | 0.7 |
| *Streptococcus spp* | CAg-CCT-ACA-ATC-CgA-ACC-gAg-ATT | 0.6 |
| *Streptococcus spp* | CAg-CCT-ACA-ATC-CgA-ACC-gAg-ACT | 0.6 |
| *Streptococcus spp* | (Cal Red 610)CgC-AAg-CCg-gTg-ACg-gCA-AgC(RTQ) | 0.5 |

# Supplementary Table 3. Average of relative abundances of vaginal microbial composition at genus level with significantly statistical difference in pregnant women with the aerobic vaginitis.

| Taxon name | The Average of Relative abundances (%) (±S.D.) | | | *p-*value | Bonferroni correction  (*p*- value) | | |
| --- | --- | --- | --- | --- | --- | --- | --- |
|  | **NF**  **(N=99)** | **AV1 (N=42)** | **AV2 (N=18)** |  | **NF:AV1** | **NF:AV2** | **AV1:AV2** |
| *Bacteria*; *Firmicutes*; *Bacilli*; *Lactobacillales*; *Lactobacillaceae*;  *Lactobacillus* | **93.441**  **(±19.411)** | **78.275**  **(±35.001)** | **55.003**  **(±37.920)** | **0.000** | 0.099 | **0.000** | 0.024 |
| *Bacteria*; *Actinobacteria*; *Actinobacteria_c*; *Bifidobacteriales*;  *Bifidobacteriaceae*; *Gardnerella* | **2.063**  **(±7.215)** | **8.439**  **(±15.741)** | **25.086**  **(±29.173)** | **0.000** | 0.140 | **0.000** | **0.004** |
| *Bacteria*; *Bacteroidetes*; *Bacteroidia*; *Bacteroidales*; *Prevotellaceae*; *Prevotella* | 0.676  (±4.518) | **3.078**  **(±7.435)** | **6.507**  **(±11.761)** | 0.067 | 0.935 | 0.018 | 0.076 |
| *Bacteria*; *Actinobacteria*; *Actinobacteria_c*; *Bifidobacteriales*;  *Bifidobacteriaceae*; *Bifidobacterium* | **1.783**  **(±11.410)** | **2.267**  **(±14.498)** | **5.545**  **(±22.841)** | 0.603 | 0.673 | 0.441 | 0.311 |
| *Bacteria*; *Actinobacteria*; *Coriobacteriia*; *Coriobacteriales*;  *Coriobacteriaceae*; *Atopobium* | 0.355  (±1.753) | **3.891**  **(±10.041)** | **4.095**  **(±11.297)** | 0.177 | 0.683 | 0.051 | 0.274 |
| *Bacteria*; *Firmicutes*; *Negativicutes*; *Veillonellales*; *Veillonellace*;  *Megasphaera* | 0.134  (±0.745) | 0.660  (±1.790) | 0.966  (±1.634) | **0.033** | 0.076 | **0.016** | 0.407 |
| *Bacteria*; *Firmicutes*; *Negativicutes*; *Veillonellales*; *Veillonellaceae*; *Dialister* | 0.054  (±0.251) | 0.422  (±1.125) | 0.896  (±1.527) | **0.010** | 0.287 | **0.002** | 0.074 |
| *Bacteria*; *Firmicutes*; *Bacilli*; *Lactobacillales*; *Aerococcaceae*;  *Aerococcus* | 0.039  (±0.353) | 0.238  (±0.666) | 0.242  (±0.453) | **0.001** | 0.026 | **0.000** | 0.161 |
| *Bacteria*; *Actinobacteria*; *Actinobacteria_c*; *Corynebacteriales*;  *CP009312_f*; *CP009312_g* | 0.002  (±0.005) | 0.000  (±0.000) | 0.000  (±0.000) | **0.020** | 0.019 | 0.121 | 1.000 |
| *Bacteria*; *Actinobacteria*; *Coriobacteriia*; *Coriobacteriales*;  *Coriobacteriaceae*; *KQ959671_g* | 0.010  (±0.065) | 0.220  (±0.642) | 0.437  (±1.011) | **0.047** | 0.038 | 0.043 | 0.695 |
| *Bacteria*; *Firmicutes*; *Bacilli*; *Bacillales*; *Staphylococcaceae*;  *Staphylococcus* | 0.002  (±0.008) | 0.004  (±0.010) | 0.002  (±0.005) | 0.452 | 0.223 | 0.572 | 0.667 |
| *Bacteria*; *Firmicutes*; *Bacilli*; *Lactobacillales*; *Streptococcaceae*;  *Streptococcus* | 0.002  (±0.008) | 0.005  (±0.021) | 0.014  (±0.044) | 0.753 | 0.876 | 0.453 | 0.576 |
| *Bacteria*; *Proteobacteria*; *Gammaproteobacteria*; *Enterobacteriales*; *Enterobacteriaceae*; *Hafnia* | 0.004  (±0.031) | 0.005  (±0.032) | 0.000  (±0.000) | 0.753 | 0.836 | 0.456 | 0.513 |
| *Bacteria*; *Proteobacteria*; *Gammaproteobacteria*; *Enterobacteriales*; *Enterobacteriaceae*; *Serratia* | 0.002  (±0.013) | 0.001  (±0.081) | 0.000  (±0.000) | 0.636 | 0.637 | 0.386 | 0.513 |
| *Bacteria*; *Proteobacteria*; *Gammaproteobacteria*; *Enterobacteriales*; *Enterobacteriaceae*; *Escherichia* | 0.001  (±0.009) | 0.000  (±0.002) | 0.000  (±0.000) | 0.503 | 0.470 | 0.332 | 0.513 |

# All relative abundance data for genus that were significantly different are presented in this table (RA > 0.001 %). Relative abundance was analyzed using the Kruskal-Wallis H test followed by the Mann-Whitney U test using Bonferroni correction to adjust the probability. Bonferroni-adjusted *p* values were used (*p* < 0.05/3). Thick letters or numbers ; OTUs with RA > 1%, *p* < 0.05 in K-W or *p* < 0.05/3 in Bonferroni correction. Blue colored letters; OTUs with the highest RA in NF group.

# Supplementary Table 4. Average of relative abundances of vaginal microbial composition at species level with significantly statistical difference in pregnant women with the aerobic vaginitis.

| Taxon name | The Average of Relative abundances (%) (±S.D.) | | | *p* -  value | Bonferroni correction  (*p*- value) | | |
| --- | --- | --- | --- | --- | --- | --- | --- |
|  | **NF (N=99)** | **AV1 (N=42)** | **AV2 (N=18)** |  | **NF:AV1** | **NF:AV2** | **AV1:AV2** |
| ***Bacteria*; *Firmicutes*; *Bacilli*; *Lactobacillales*; *Lactobacillaceae*;**  ***Lactobacillus*; *Lactobacillus crispatus*** | **62.300**  **(±45.182)** | **33.934**  **(±45.454)** | 0.062  (±0.065) | **0.000** | **0.001** | **0.000** | **0.004** |
| ***Bacteria*; *Actinobacteria*; *Actinobacteria_c*; *Bifidobacteriales*;**  ***Bifidobacteriaceae*; *Gardnerella*; *ADEP_s*** | **1.542**  **(±6.017)** | **4.765**  **(±9.895)** | **17.988**  **(±22.666)** | **0.003** | 0.505 | **0.001** | **0.014** |
| ***Bacteria*; *Actinobacteria*; *Actinobacteria_c*; *Bifidobacteriales*;**  ***Bifidobacteriaceae*; *Gardnerella*; *ADET_s*** | 0.521  (±3.831) | **3.674**  **(±10.703)** | **7.098**  **(±22.690)** | **0.020** | 0.094 | **0.008** | 0.311 |
| *Bacteria*; *Actinobacteria*; *Coriobacteriia*; *Coriobacteriales*;  *Coriobacteriaceae*; *Atopobium*; *Atopobium vaginae* | 0.354  (±1.678) | **3.890**  **(±10.041)** | **4.094**  **(±11.297)** | 0.176 | 0.679 | 0.051 | 0.274 |
| *Bacteria*; *Bacteroidetes*; *Bacteroidia*; *Bacteroidales*; *Prevotellaceae*; *Prevotella*; *Prevotella amnii* | 0.002  (±0.008) | **1.362**  **(±5.315)** | **1.729**  **(±3.991)** | 0.515 | 0.808 | 0.245 | 0.435 |
| *Bacteria*; *Bacteroidetes*; *Bacteroidia*; *Bacteroidales*; *Prevotellaceae*; *Prevotella*; *Prevotella bivia* | 0.024  (±0.103) | 0.840  (±3.478) | **2.294**  **(±9.416)** | 0.996 | 0.986 | 0.910 | 0.992 |
| *Bacteria*; *Firmicutes*; *Negativicutes*; *Veillonellales*; *Veillonellace:*  *Megasphaera*; *ADGP_s* | 0.133  (±0.745) | 0.652  (±1.778) | 0.966  (±1.634) | **0.009** | 0.023 | **0.005** | 0.407 |
| *Bacteria*; *Firmicutes*; *Negativicutes*; *Veillonellales*; *Veillonellaceae*; *Dialister*; *KQ960846_s* | 0.010  (±0.038) | 0.271  (±0.978) | 0.584  (±1.298) | **0.006** | 0.090 | **0.002** | 0.127 |
| *Bacteria*; *Actinobacteria*; *Coriobacteriia*; *Coriobacteriales*;  *Coriobacteriaceae*; *KQ959671_g*; *KQ959671_s* | 0.010  (±0.065) | 0.220  (±0.642) | 0.437  (±1.011) | **0.047** | 0.038 | 0.043 | 0.695 |
| *Bacteria*; *Fusobacteria*; *Fusobacteria_c*; *Fusobacteriaes*;  *Leptotrichiaceae*; *Sneathia*; *Sneathia sanguinegens* | 0.001  (±0.007) | 0.396  (±1.955) | 0.132  (±0.307) | **0.008** | 0.091 | **0.001** | 0.230 |
| *Bacteria*; *Firmicutes*; *Bacilli*; *Lactobacillales*; *Aerococcacea*; *Aerococcus*; *Aerococcus christensenii* | 0.039  (±0.353) | 0.238  (±0.666) | 0.242  (±0.453) | **0.001** | 0.026 | **0.000** | 0.161 |
| *Bacteria*; *Firmicutes*; *Negativicutes*; *Veillonellales*;  *Veillonellaceae*; *Dialister*; *Dialister micraerophilus* | 0.043  (±0.238) | 0.150  (±0.367) | 0.312  (±0.650) | **0.002** | 0.169 | **0.000** | 0.064 |
| *Bacteria*; *Firmicutes*; *Bacilli*; *Lactobacillales*; *Lactobacillaceae*;  *Lactobacillus*; *Lactobacillus reuteri* | 0.090  (±0.221) | 0.026  (±0.072) | 0.043  (±0.125) | **0.038** | 0.049 | 0.060 | 0.568 |
| *Bacteria*; *Actinobacteria*; *Actinobacteria_c*; *Corynebacteriales*;  *CP009312_f*; *CP009312_g*; *CP009312_s* | 0.002  (±0.005) | 0.000  (±0.000) | 0.000  (±0.000) | **0.020** | 0.019 | 0.121 | 1.000 |

# All relative abundance data for species that were significantly different are presented in this table (RA > 0.001 %). Relative abundance was analyzed using the Kruskal-Wallis H test followed by the Mann-Whitney U test using Bonferroni correction to adjust the probability. Bonferroni-adjusted *p* values were used (*p* < 0.05/3). Thick letter or numbers ; OTUs with RA > 1%, *p* < 0.05 in K-W or *p* < 0.05/3 in Bonferroni correction. Blue colored letters ; OTUs with the highest RA in NF group.

# Supplement Table 5. Average of relative abundances of vaginal microbial composition with significantly statistical difference by *Lactobacillary* grade criteria in pregnant women with the aerobic vaginitis.

| Taxon name | The average of Relative abundances (%) (±S.D.) | | | *p*- value | Bonferroni correction  (*p*- value) | | |
| --- | --- | --- | --- | --- | --- | --- | --- |
|  | **Grade 0 (n=120)** | **Grade 1 (n=22)** | **Grade 2 (n=17)** |  | **G0:G1** | **G0:G2** | **G1:G2** |
| ***Bacteria*; *Firmicutes*; *Bacilli*; *Lactobacillales*; *Lactobacillaceae*;**  ***Lactobacillus*; *Lactobacillus crispatus*** | **60.023 (±46.016)** | **17.595 (±38.031)** | 0.250 (±0.266) | **0.000** | **0.000** | **0.000** | 0.547 |
| ***Bacteria*; *Actinobacteria*; *Actinobacteria_c*; *Bifidobacteriales*;**  ***Bifidobacteriaceae*; *Gardnerella*; *ADEP_s*** | **1.841 (±8.404)** | **10.118 (±18.016)** | **13.709 (±13.422)** | **0.000** | 0.043 | **0.000** | 0.255 |
| ***Bacteria*; *Actinobacteria*; *Coriobacteriia*; *Coriobacteriales*;**  ***Coriobacteriaceae*; *Atopobium*; *Atopobium vaginae*** | 0.084 (±0.524) | **4.721 (±12.307)** | **9.242 (±12.755)** | **0.000** | **0.009** | **0.000** | 0.013 |
| ***Bacteria*; *Actinobacteria*; *Actinobacteria_c*; *Bifidobacteriales*;**  ***Bifidobacteriaceae*; *Bifidobacterium*; *Bifidobacterium_breve*** | 0.006 (±0.025) | 0.000 (±0.002) | **20.807 (±39.017)** | **0.000** | 0.699 | **0.000** | 0.087 |
| ***Bacteria*; *Actinobacteria*; *Actinobacteria_c*; *Bifidobacteriales*;**  ***Bifidobacteriaceae*; *Gardnerella*; *ADET_s*** | 0.798 (±4.483) | **8.851 (±24.341)** | **2.543 (±4.251)** | **0.000** | **0.001** | **0.000** | 0.812 |
| ***Bacteria*; *Bacteroidetes*; *Bacteroidia*; *Bacteroidales*;**  ***Prevotellaceae*; *Prevotella*; *Prevotella amnii*** | 0.002 (±0.008) | 0.251 (±1.175) | **4.872 (±8.555)** | **0.000** | 0.661 | **0.000** | 0.092 |
| *Bacteria*; *Actinobacteria*; *Actinobacteria_c*; *Bifidobacteriales*;  *Bifidobacteriaceae*; *Bifidobacterium*; *Bifidobacterium_longum* | 0.141 (±1.539) | 0.001 (±0.005) | 0.004 (±0.012) | **0.023** | 0.180 | **0.004** | 0.685 |
| ***Bacteria*; *Bacteroidetes*; *Bacteroidia*; *Bacteroidales*;**  ***Prevotellaceae*; *Prevotella*; *Prevotella bivia*** | 0.012 (±0.037) | 0.660 (±2.269) | **3.707 (**±10.835) | **0.015** | 0.897 | **0.004** | 0.124 |
| ***Bacteria*; *Firmicutes*; *Negativicutes*; *Veillonellales*; *Veillonellaceae*; *Megasphaera*; *ADGP_s*** | 0.091 (±0.691) | 0.445 (±1.392) | **2.194 (±2.339)** | **0.000** | 0.021 | **0.000** | **0.004** |
| ***Bacteria*; *Firmicutes*; *Clostridia*; *Clostridiales*; *Ruminococcaceae*;**  ***KQ959578_g*; *AY958888_s*** | 0.001 (±0.009) | 0.343 (±1.610) | **2.331 (±4.964)** | **0.000** | 0.751 | **0.000** | **0.012** |
| ***Bacteria*; *Bacteroidetes*; *Bacteroidia*; *Bacteroidales*; *Prevotellaceae*; *Prevotella*; *Prevotella timonensis*** | 0.036 (±0.181) | 0.189 (±0.725) | **2.663 (±9.910)** | **0.016** | 0.556 | **0.004** | 0.172 |
| ***Bacteria*; *Fusobacteria*; *Fusobacteria_c*; *Fusobacteriales*;**  ***Leptotrichiaceae*; *Sneathia*; *Leptotrichia amnionii*** | 0.012 (±0.104) | 0.789 (±3.231) | **2.391 (±9.055)** | **0.001** | 0.215 | **0.000** | 0.279 |
| *Bacteria*; *Firmicutes*; *Negativicutes*; *Veillonellales*; *Veillonellaceae*;  *Dialister*; *KQ960846_s* | 0.004 (±0.015) | 0.340 (±1.094) | 0.878 (±1.562) | **0.000** | **0.008** | **0.000** | **0.014** |
| *Bacteria*; *Actinobacteria*; *Coriobacteriia*; *Coriobacteriales*;  *Coriobacteriaceae*; *KQ959671_g*; *KQ959671_s* | 0.001 (±0.004) | 0.207 (±0.690) | 0.790 (±1.119) | **0.000** | 0.019 | **0.000** | 0.023 |
| ***Bacteria*; *Fusobacteria*; *Fusobacteria_c*; *Fusobacteriales*;**  ***Leptotrichiaceae*; *Sneathia*; *Sneathia sanguinegens*** | 0.001 (±0.007) | 0.049 (±0.172) | **1.053** (±3.058) | **0.000** | 0.112 | **0.000** | 0.146 |
| *Bacteria*; *Firmicutes*; *Bacilli*; *Lactobacillales*; *Aerococcaceae*;  *Aerococcus*; *Aerococcus christensenii* | 0.031 (±0.322) | 0.170 (±0.485) | 0.629 (±0.916) | **0.000** | **0.000** | **0.000** | 0.029 |
| ***Bacteria*; *Firmicutes*; *Tissierellia*; *Tissierellales*; *Peptoniphilaceae*; *Parvimonas*; *KQ959647_s*** | 0.001 (±0.006) | 0.022 (±0.078) | **1.107** (±2.343) | **0.000** | 0.293 | **0.000** | 0.077 |
| *Bacteria*; *Firmicutes*; *Negativicutes*; *Veillonellales*; *Veillonellaceae*;  *Dialister*; *Dialister micraerophilus* | 0.010 (±0.053) | 0.306 (±0.678) | 0.487 (±0.595) | **0.000** | **0.006** | **0.000** | **0.004** |
| ***Bacteria*; *Bacteroidetes*; *Bacteroidia*; *Bacteroidales*; *Prevotellaceae*; *Prevotella*; *Prevotella melaninogenica*** | 0.000 (±0.003) | 0.000 (±0.000) | **1.072 (±4.116)** | **0.000** | 0.455 | **0.000** | 0.221 |
| *Bacteria*; *Firmicutes*; *Bacilli*; *Lactobacillales*; *Lactobacillaceae*;  *Lactobacillus*; *Lactobacillus reuteri* | 0.080 (±0.205) | 0.053 (±0.129) | 0.000 (±0.000) | **0.010** | 0.202 | **0.004** | 0.347 |
| *Bacteria*; *Tenericutes*; *Mollicutes*; *Mycoplasmatales*; *Mycoplasmataceae*; *Mycoplasma_g4*; *Mycoplasma hominis* | 0.057 (±0.622) | 0.208 (±0.857) | 0.009 (±0.024) | **0.001** | **0.015** | **0.000** | 0.726 |
| *Bacteria*; *Actinobacteria*; *Actinobacteria_c*; *Actinomycetales*; *Actinomycetaceae*; *Mobiluncus*; *Mobiluncus mulieris* | 0.000 (±0.000) | 0.212  (±0.996) | 0.000 (±0.000) | **0.044** | 0.020 | 1.000 | 0.812 |
| *Bacteria*; *Firmicutes*; *Tissierellia*; *Tissierellales*; *Peptoniphilaceae*; *Peptoniphilus*; *KQ960236_s* | 0.003 (±0.010) | 0.062 (±0.275) | 0.096 (±0.210) | **0.044** | 0.523 | **0.020** | 0.200 |
| *Bacteria*; *Firmicutes*; *Bacilli*; *Lactobacillales*; *Lactobacillaceae*; *Lactobacillus*; *Lactobacillus casei* | 0.000 (±0.000) | 0.000 (±0.000) | 0.243 (±1.000) | **0.015** | 1.000 | **0.008** | 0.769 |
| *Bacteria*; *Firmicutes*; *Tissierellia*; *Tissierellales*; *Peptoniphilaceae*; *Anaerococcus*; *Anaerococcus tetradius* | 0.003 (±0.014) | 0.018 (±0.059) | 0.082 (±0.229) | **0.005** | 0.176 | **0.001** | 0.392 |
| *Bacteria*; *Bacteroidetes*; *Bacteroidia*; *Bacteroidales*; *EU845084_f*; *GU302773_g*; *HM123928_s* | 0.000 (±0.000) | 0.000 (±0.000) | 0.133 (±0.548) | **0.015** | 1.000 | **0.008** | 0.769 |
| *Bacteria*; *Bacteroidetes*; *Bacteroidia*; *Bacteroidales*; *Porphyromonadaceae*; *Porphyromonas*; *Porphyromonas asaccharolytica* | 0.000 (±0.001) | 0.001 (±0.004) | 0.118 (±0.409) | **0.001** | 0.172 | **0.000** | 0.492 |
| *Bacteria*; *Actinobacteria*; *Coriobacteriia*; *Coriobacteriales*; *Coriobacteriaceae*; *Atopobium*; *AEDQ_s* | 0.000 (±0.000) | 0.000 (±0.000) | 0.072 (±0.298) | **0.015** | 1.000 | **0.008** | 0.769 |
| *Bacteria*; *Firmicutes*; *Clostridia*; *Clostridiales*; *Lachnospiraceae*;  *Howardella*; *AF385567_s* | 0.001 (±0.006) | 0.009 (±0.022) | 0.004 (±0.010) | **0.030** | 0.021 | 0.045 | 0.922 |
| *Bacteria*; *Firmicutes*; *Bacilli*; *Bacillales*; *Gemella_f*; *Gemella*;  *Gemella asaccharolytica* | 0.000 (±0.000) | 0.008 (±0.036) | 0.000 (±0.000) | **0.044** | 0.020 | 1.000 | 0.812 |
| *Bacteria*; *Firmicutes*; *Erysipelotrichi*; *Erysipelotrichales*; *Erysipelotrichaceae*; *Bulleidia*; *Bulleidia_extructa* | 0.000 (±0.000) | 0.001 (±0.003) | 0.016 (±0.059) | **0.002** | 0.020 | **0.000** | 0.685 |
| *Bacteria*; *Actinobacteria*; *Actinobacteria_c*; *Actinomycetales*; *Actinomycetaceae*; *Mobiluncus*; *Mobiluncus curtisii* | 0.000 (±0.003) | 0.000 (±0.000) | 0.009 (±0.021) | **0.000** | 0.543 | **0.000** | 0.221 |
| *Bacteria*; *Actinobacteria*; *Actinobacteria_c*; *Bifidobacteriales*; *Bifidobacteriaceae*; *Bifidobacterium*; *Bifidobacterium commune* | 0.000 (±0.000) | 0.001 (±0.006) | 0.008 (±0.018) | **0.000** | 0.020 | **0.000** | 0.475 |
| *Bacteria*; *Proteobacteria*; *Alphaproteobacteria*; *Sphingomonadales*; *Sphingomonadaceae*; *Sphingomonas*; *Sphingomonas desiccabilis* | 0.000 (±0.000) | 0.001 (±0.004) | 0.000 (±0.000) | **0.044** | 0.020 | 1.000 | 0.812 |
| *Bacteria*; *Bacteroidetes*; *Bacteroidia*; *Bacteroidales*; *Bacteroidaceae*; *Bacteroides*; *Bacteroides uniformis* | 0.000 (±0.000) | 0.000 (±0.000) | 0.003 (±0.013) | **0.015** | 1.000 | **0.008** | 0.769 |

# All relative abundance data for species that were significantly different are presented in this table (RA > 0.001 %). Relative abundance was analyzed using the Kruskal-Wallis H test followed by the Mann-Whitney *U* test using Bonferroni correction to adjust the probability. Bonferroni-adjusted *p* values were used (*p* < 0.05/3). Thick letters or numbers ; OTUs with RA > 1%, *p* < 0.05 in K-W or *p* < 0.05/3 in Bonferroni correction. Blue colored letters ; OTUs with the highest RAs of OTUs in Grade 0, other OTUs ; black letters .

# Supplementary Table 6. Average of relative abundances of vaginal microbial composition with significantly statistical difference by toxic leukocyte grade in pregnant women with aerobic vaginitis.

| Taxon name | The average of Relative abundances (%) (±S.D.) | | | *p-*value | Bonferroni correction (*p*- value) | | |
| --- | --- | --- | --- | --- | --- | --- | --- |
|  | Grade 0 (N=102) | Grade 1 (N=45) | Grade 2 (N=12) |  | G0:G1 | G0:G2 | G1:G2 |
| ***Bacteria*; *Firmicutes*; *Bacilli*; *Lactobacillales*; *Lactobacillaceae*;**  ***Lactobacillus*; *Lactobacillus crispatus*** | **51.260 (±47.503)** | **42.663 (±48.416)** | **37.138 (±46.674)** | 0.247 | 0.197 | 0.175 | 0.984 |
| ***Bacteria*; *Firmicutes*; *Bacilli*; *Lactobacillales*; *Lactobacillaceae*;**  ***Lactobacillus*; *Lactobacillus iners*** | **25.053 (±38.067)** | **32.864 (±43.615)** | **35.904 (±42.136)** | 0.761 | 0.893 | 0.419 | 0.652 |
| ***Bacteria*; *Actinobacteria*; *Actinobacteria_c*; *Bifidobacteriales*; *Bifidobacteriaceae*; *Gardnerella*; *ADEP­_s*** | **3.158 (±8.188)** | **5.004 (±14.137)** | **10.770 (±21.012)** | 0.184 | 0.108 | 0.512 | 0.135 |
| ***Bacteria*; *Actinobacteria*; *Coriobacteriia*; *Coriobacteriales*; *Coriobacteriaceae*; *Atopobium*; *Atopobium vaginae*** | **1.656 (±6.166)** | **2.125 (±8.804)** | **0.538 (±1.445)** | **0.035** | **0.016** | 0.224 | 0.926 |
| ***Bacteria*; *Firmicutes*; *Bacilli*; *Lactobacillales*; *Lactobacillaceae*;**  ***Lactobacillus*; *Lactobacillus fornicalis*** | **3.127 (±14.832)** | **0.001 (±0.003)** | **0.001 (±0.003)** | **0.049** | 0.031 | 0.169 | 0.826 |
| *Bacteria*; *Bacteroidetes*; *Bacteroidia*; *Bacteroidales*; *Prevotellaceae*;  *Prevotella*; *Prevotella amnii* | 0.791 (±3.827) | 0.000 (±0.000) | 0.657 (±2.276) | **0.025** | **0.007** | 0.587 | 0.053 |
| *Bacteria*; *Firmicutes*; *Clostridia*; *Clostridiales*; *Ruminococcaceae*;  *KQ959578_g*; *AY958888_s* | 0.459 (±2.273) | 0.000 (±0.000) | 0.038 (±0.131) | **0.044** | **0.013** | 0.668 | 0.053 |
| *Bacteria*; *Bacteroidetes*; *Bacteroidia*; *Bacteroidales*; *Prevotellaceae*;  *Prevotella*; *Prevotella timonensis* | 0.522 (±4.078) | 0.011 (±0.034) | 0.001 (±0.003) | **0.012** | 0.017 | 0.047 | 0.456 |
| *Bacteria*; *Proteobacteria*; *Gammaproteobacteria*; *Pseudomonaales*;  *Pseudomonadaceae*; *Pseudomonas*; *Pseudomonas veronii* | 0.003 (±0.028) | 0.159 (±0.707) | 0.294 (±1.003) | **0.001** | **0.004** | **0.000** | 0.218 |
| *Bacteria*; *Bacteroidetes*; *Sphingobacteriia*; *Sphingobacteriales*; *Sphingobacteriaceae*; *Sphingobacterium*; *Sphingobacterium multivorum* | 0.002 (±0.016) | 0.077 (±0.343) | 0.146 (±0.497) | **0.013** | 0.049 | **0.001** | 0.300 |
| *Bacteria*; *Proteobacteria*; *Alphaproteobacteria*; *Rhizobiales*;  *Brucellaceae*; *Ochrobactrum*; *Ochrobactrum intermedium* | 0.001 (±0.015) | 0.068 (±0.318) | 0.137 (±0.455) | **0.010** | **0.015** | **0.001** | 0.440 |
| *Bacteria*; *Firmicutes*; *Bacilli*; *Bacillales*; *Bacillaceae*;  *Bacillus*; *CM000739_s* | 0.000 (±0.000) | 0.011 (±0.051) | 0.039 (±0.127) | **0.000** | **0.002** | **0.000** | 0.145 |
| *Bacteria*; *Proteobacteria*; *Gammaproteobacteria*; *Enterobacteriales*;  *Enterobacteriaceae*; *Hafnia*; *Obesumbacterium proteus* | 0.000 (±0.000) | 0.009 (±0.046) | 0.017 (±0.060) | **0.025** | **0.009** | **0.004** | 0.825 |
| *Bacteria*; *Proteobacteria*; *Betaproteobacteria*; *Burkholderiales*; *Ralstonia_f*; *Ralstonia*; *Ralstonia pickettii* | 0.009 (±0.027) | 0.000 (±0.003) | 0.000 (±0.000) | **0.009** | **0.008** | 0.105 | 0.606 |
| *Bacteria; Actinobacteria; Actinobacteria_c; Corynebacteriales; Nocardiaceae; Rhodococcus; Rhodococcus erythropolis* | 0.000 (±0.000) | 0.001 (±0.005) | 0.002 (±0.008) | **0.041** | 0.132 | **0.004** | 0.326 |
| *Bacteria*; *Firmicutes*; *Negativicutes*; *Veillonellales*; *Veillonellaceae*;  *Dialister*; *Dialister propionicifaciens* | 0.001 (±0.010) | 0.000 (±0.000) | 0.003 (±0.008) | **0.040** | 0.132 | 0.105 | **0.006** |
| *Bacteria*; *Firmicutes*; *Bacilli*; *Lactobacillales*; *Streptococcaceae*;  *Streptococcus*; *JYGU_s* | 0.000 (±0.001) | 0.001 (±0.005) | 0.002 (±0.005) | **0.010** | **0.015** | **0.001** | 0.440 |
| *Bacteria*; *Firmicutes*; *Bacilli*; *Lactobacillales*;  *Streptococcaceae*; *Streptococcus*; *CP006776_s* | 0.000 (±0.001) | 0.002 (±0.006) | 0.001 (±0.004) | **0.045** | **0.014** | 0.065 | 0.921 |
| *Bacteria*; *Firmicutes*; *Bacilli*; *Lactobacillales*; *Enterococcaceae*;  *Vagococcus*; *Vagococcus salmoninarum* | 0.000 (±0.000) | 0.001 (±0.004) | 0.002 (±0.006) | **0.045** | 0.169 | **0.004** | 0.595 |
| *Bacteria*; *Proteobacteria*; *Gammaproteobacteria*; *Pseudomonadales*;  *Pseudomonadaceae*; *Pseudomonas*; *Pseudomonas chlororaphis* | 0.000 (±0.000) | 0.002 (±0.007) | 0.001 (±0.004) | **0.025** | **0.009** | **0.004** | 0.860 |

# All relative abundance data for species that were significantly different are presented in this table (RA > 0.001 %). Relative abundance was analyzed using the Kruskal-Wallis H test followed by the Mann-Whitney *U* test using Bonferroni correction to adjust the probability. Bonferroni-adjusted *p* values were used (*p* < 0.05/3). Thick letters and numbers ; OTUs with RA > 1%, *p* < 0.05 in K-W or *p* < 0.05/3 in Bonferroni correction. Blue colored letters ; OTUs with the highest RAs in Grade 0, other OTUs ; black letters.

# Supplementary Table 7. Average of relative abundances of vaginal microbial composition with significantly statistical difference by White Blood Cell grade in pregnant women with aerobic vaginitis.

| Taxon name | The average of Relative abundances (%) (±S.D.) | | | *p-value* | Bonferroni correction (*p*- value) | | |
| --- | --- | --- | --- | --- | --- | --- | --- |
|  | **Grade 0**  **(N=37)** | **Grade 1**  **(N=71)** | **Grade 2**  **(N=51)** |  | **G0:G1** | **G0:G2** | **G1:G2** |
| ***Bacteria*; *Firmicutes*; *Bacilli*; *Lactobacillales*; *Lactobacillaceae*;**  ***Lactobacillus*; *Lactobacillus crispatus*** | **53.459 (±46.342)** | **57.982 (±47.732)** | **29.398 (±43.857)** | **0.000** | 0.183 | **0.005** | **0.000** |
| ***Bacteria*; *Firmicutes*; *Bacilli*; *Lactobacillales*; *Lactobacillaceae*;**  ***Lactobacillus*; *Lactobacillus iners*** | **23.873 (±37.910)** | **22.641 (±37.570)** | **38.711 (±43.178)** | **0.033** | 0.243 | 0.166 | **0.012** |
| ***Bacteria*; *Actinobacteria*; *Coriobacteriia*; *Coriobacteriales***  **; *Coriobacteriaceae* ; *Atopobium*; *Atopobium vaginae*** | 0.999 (±2.870) | **1.510 (±6.073)** | **2.486 (±9.345)** | **0.020** | **0.005** | 0.101 | 0.387 |
| *Bacteria*; *Firmicutes*; *Clostridia*; *Clostridiales*; *Ruminococcaceae*;  *KQ959578_g*; *AY958888_s* | 0.958 (±3.505) | 0.046 (±0.374) | 0.169 (±1.059) | **0.036** | **0.012** | 0.124 | 0.385 |
| *Bacteria*; *Proteobacteria*; *Gammaproteobacteria*; *Pseudomonadales*;  *Pseudomonadaceae*; *Pseudomonas*; *Pseudomonas guariconensis* | 0.005 (±0.013) | 0.006 (±0.027) | 0.000 (±0.000) | **0.037** | 0.300 | **0.007** | 0.054 |
| *Bacteria*; *Firmicutes*; *Clostridia*; *Clostridiales*; *Ruminococcaceae*;  *Mageeibacillus*; *Mageeibacillus indolicus* | 0.204 (±1.227) | 0.000 (±0.000) | 0.002 (±0.014) | **0.008** | **0.005** | 0.082 | 0.238 |
| *Bacteria*; *Proteobacteria*; *Betaproteobacteria*; *Neisseriales*;  *Neisseriaceae*; *Neisseria*; *AY064548_s* | 0.004 (±0.020) | 0.000 (±0.000) | 0.000 (±0.000) | **0.036** | 0.049 | 0.095 | 1.000 |
| *Bacteria*; *Firmicutes*; *Bacilli*; *Bacillales*; *Staphylococcaceae*  ; *Staphylococcus*; *Staphylococcus cohnii* | 0.000 (±0.000) | 0.000 (±0.001) | 0.002 (±0.007) | **0.018** | 0.207 | 0.020 | 0.050 |
| *Bacteria*; *Firmicutes*; *Tissierellia*; *Tissierellales*; *Peptoniphilaceae*;  *Ezakiella*; *Ezakiella peruensis* | 0.004 (±0.017) | 0.000 (±0.000) | 0.000 (±0.000) | **0.036** | 0.049 | 0.095 | 1.000 |
| *Bacteria*; *Proteobacteria*; *Betaproteobacteria*; *Burkholderiales*;  *Sutterellaceae*; *Sutterella*; *HQ751400_s* | 0.002 (±0.005) | 0.000 (±0.003) | 0.000 (±0.000) | **0.029** | 0.093 | 0.017 | 0.229 |

# All relative abundance data for species that were significantly different are presented in this table (RA > 0.001 %). Relative abundance was analyzed using the Kruskal-Wallis H test followed by the Mann-Whitney *U* test using Bonferroni correction to adjust the probability. Bonferroni-adjusted *p* values were used (*p* < 0.05/3). Thick letters and numbers ; OTUs with RA > 1%, *p* < 0.05 in K-W or *p* < 0.05/3 in Bonferroni correction. Blue colored letters ; OTUs with the highest RAs in Grade 0, other OTUs ; black letters

# Supplementary Table 8. Average of relative abundances of vaginal microbial composition with significantly statistical difference by Background flora grade in pregnant women with aerobic vaginitis.

| Taxon name | The average of Relative abundances (%) (±S.D.) | | | *p*-value | Bonferroni correction  (*p*- value) | | |
| --- | --- | --- | --- | --- | --- | --- | --- |
|  | **Grade 0 (N=107)** | **Grade 1 (N=40)** | **Grade 2 (N=12)** |  | **G0:G1** | **G0:G2** | **G1:G2** |
| ***Bacteria***; ***Firmicutes***; ***Bacilli***; ***Lactobacillales***; ***Lactobacillaceae***;  ***Lactobacillus***; ***Lactobacillus crispatus*** | **56.058 (±46.998)** | **37.688 (±47.171)** | **7.363 (±24.985)** | **0.000** | **0.003** | **0.000** | 0.307 |
| ***Bacteria***; ***Actinobacteria***; ***Actinobacteria_c***; ***Bifidobacteriales***;  ***Bifidobacteriaceae***; ***Gardnerella***; ***ADEP_s*** | **3.029 (±9.756)** | **2.059 (±5.739)** | **22.506 (±22.061)** | **0.001** | 0.867 | **0.000** | **0.001** |
| ***Bacteria***; ***Actinobacteria***; ***Coriobacteriia***; ***Coriobacteriales***;  ***Coriobacteriaceae***; ***Atopobium***; ***Atopobium vaginae*** | **1.414 (±6.00)** | **1.067 (±5.237)** | **6.421 (±13.877)** | **0.001** | 0.071 | **0.002** | **0.000** |
| ***Bacteria***; ***Actinobacteria***; ***Actinobacteria_c***; ***Bifidobacteriales***;  ***Bifidobacteriaceae***; ***Gardnerella***; ***ADET_s*** | **0.900 (±4.579)** | **2.483 (±10.194)** | **11.503 (±28.346)** | **0.002** | 0.781 | **0.000** | **0.008** |
| ***Bacteria***; ***Firmicutes***; ***Bacilli***; ***Lactobacillales***; ***Lactobacillaceae***;  ***Lactobacillus***; ***Lactobacillus acidophilus*** | **0.000 (±0.000)** | **1.625 (±10.280)** | **2.644 (±9.158)** | **0.037** | 0.102 | **0.003** | 0.379 |
| ***Bacteria***; ***Firmicutes***; ***Negativicutes***; ***Veillonellales***; ***Veillonellaceae***; ***Megasphaera***; ***ADGP_s*** | **0.236 (±1.109)** | **0.286 (±0.976)** | **1.769 (±2.292)** | **0.000** | 0.973 | **0.000** | **0.001** |
| *Bacteria*; *Firmicutes*; *Negativicutes*; *Veillonellales*; *Veillonellaceae*; *Dialister*; *KQ960846_s* | 0.020 (±0.094) | 0.279 (±1.017) | 0.796 (±1.590) | **0.000** | 0.496 | **0.000** | **0.001** |
| *Bacteria*; *Actinobacteria*; *Coriobacteriia*; *Coriobacteriales*; *Coriobacteriaceae*; *KQ959671_g*; *KQ959671_s* | 0.043 (±0.201) | 0.186 (±0.668) | 0.503 (±1.178) | **0.005** | 0.526 | **0.001** | 0.026 |
| *Bacteria*; *Fusobacteria*; *Fusobacteria_c*; *Fusobacteriales*; *Leptotrichiaceae*; *Sneathia*; *Sneathia sanguinegens* | 0.124 (±1.220) | 0.089 (±0.435) | 0.188 (±0.378) | **0.027** | 0.479 | **0.006** | 0.096 |
| *Bacteria*; *Firmicutes*; *Bacilli*; *Lactobacillales*; *Aerococcaceae*; *Aerococcus*; *Aerococcus christensenii* | 0.082 (±0.485) | 0.072 (±0.279) | 0.538 (±0.727) | **0.000** | 0.330 | **0.000** | **0.006** |
| *Bacteria*; *Firmicutes*; *Negativicutes*; *Veillonellales*; *Veillonellaceae*; *Dialister*; *Dialister micraerophilus* | 0.068 (±0.278) | 0.059 (±0.234) | 0.541 (±0.814) | **0.000** | 0.736 | **0.000** | **0.000** |
| *Bacteria*; *Proteobacteria*; *Betaproteobacteria*; *Burkholderiales*;  *Ralstonia_f*; *Ralstonia*; *Ralstonia pickettii* | 0.008 (±0.027) | 0.000 (±0.000) | 0.000 (±0.000) | **0.004** | **0.003** | 0.104 | 1.000 |
| *Bacteria*; *Firmicutes*; *Tissierellia*; *Tissierellales*; *Peptoniphilaceae*;  *Anaerococcus*; *Anaerococcus lactolyticus* | 0.000  (±0.002) | 0.004 (±0.011) | 0.000 (±0.000) | **0.001** | **0.000** | 0.738 | 0.159 |

# All relative abundance data for species that were significantly different are presented in this table (RA > 0.001 %). Relative abundance was analyzed using the Kruskal-Wallis H test followed by the Mann-Whitney *U* test using Bonferroni correction to adjust the probability. Bonferroni-adjusted *p* values were used (*p* < 0.05/3). Thick letters and numbers ; OTUs with RA > 1%, *p* < 0.05 in Kruskal-Wallis H test or *p* < 0.05/3 in Bonferroni correction. Blue colored letters ; OTUs with the highest RAs in Grade 0, other OTUs ; black letters.

# Supplementary Table 9. The comparison of relative abundances of vaginal microbiota composition between moderate aerobic vaginitis group without bacterial vaginosis (BV) (Moderate AV-1) and moderate AV group with BV (Moderate AV-2).

| Taxon name | Moderate AV-1 (N=9) | Moderate AV-2 (N=9) | *p-*value |
| --- | --- | --- | --- |
|  | Mean + S.D (range) | Mean + S.D (range) |  |
| *Bacteria*; *Firmicutes*; *Bacilli*; *Lactobacillales*; *Lactobacillaceae*;  *Lactobacillus*;  *Lactobacillus crispatus* | 0.047+0.054  (0.000 ~ 0.137) | 0.077+0.079  (0.000 ~ 0.265) | 0.297 |
| *Bacteria*; *Firmicutes*; *Bacilli*; *Lactobacillales*; *Lactobacillaceae*;  *Lactobacillus*; *Lactobacillus iners* | 57.692+47.363  (0.076 ~ 99.895) | 35.824+29.350  (0.000 ~ 77.444) | 0.222 |
| *Bacteria*; *Actinobacteria*; *Actinobacteria_c*; *Bifidobacteriales*;  *Bifidobacteriaceae*; *Gardnerella*; *ADEP_s* | 19.716+29.642  (0.000 ~ 67.178) | 16.261+16.449  (0.000 ~ 38.673) | 1.000 |
| *Bacteria*; *Actinobacteria*; *Actinobacteria_c*; *Bifidobacteriales*;  *Bifidobacteriaceae*; *Gardnerella*; *ADET_s* | 0.003+0.008  (0.000 ~ 0.025) | 14.193+32.329  (0.008 ~ 99.834) | **0.001** |
| *Bacteria*; *Bacteroidetes*; *Bacteroidia*; *Bacteroidales*; *Prevotellaceae*; *Prevotella*; *Prevotella bivia* | 0.004+0.008  (0.000 ~ 0.020) | 4.584+13.700  (0.000 ~ 41.118) | 0.666 |
| *Bacteria*; *Actinobacteria*; *Coriobacteriia*; *Coriobacteriales* ;  *Coriobacteriaceae*; *Atopobium*; *Atopobium vaginae* | 5.513+16.538  (0.000 ~ 49.615) | 2.676+3.014  (0.000 ~ 8.888) | **0.040** |
| *Bacteria*; *Firmicutes*; *Negativicutes*; *Veillonellales*;  *Veillonellaceae*; *Megasphaera*; *ADGP_s* | 0.000 | 1.931+1.976  (0.000 ~ 5.003) | **0.014** |
| *Bacteria*; *Firmicutes*; *Negativicutes*; *Veillonellales*;  *Veillonellaceae*; *Dialister*; *KQ960846_s* | 0.001+0.002  ( 0.000 ~ 0.005 ) | 1.167+1.739  (0.000 ~ 5.540) | **0.006** |
| *Bacteria*; *Bacteroidetes*; *Bacteroidia*; *Bacteroidales*;  *Prevotellaceae*; *Prevotella*; *Prevotella buccalis* | 0.000 | 4.653+11.087  (0.000 ~ 33.216) | 0.436 |
| *Bacteria*; *Bacteroidetes*; *Bacteroidia*; *Bacteroidales*; *Prevotellaceae*; *Prevotella*; *Prevotella amnii* | 0.000 | 3.458+5.396  (0.000 ~ 13.672) | 0.258 |
| *Bacteria*; *Firmicutes*; *Bacilli*; *Lactobacillales*;  *Aerococcaceae*; *Aerococcus*; *Aerococcus christensenii* | 0.006+0.018  (0.000 ~ 0.055) | 0.478+0.579  (0.000 ~ 1.648) | **0.024** |
| *Bacteria*; *Firmicutes*; *Negativicutes*; *Veillonellales*;  *Veillonellac-eae*; *Dialister*; *Dialister micraerophilus* | 0.292+0.871  (0.000 ~ 2.615) | 0.332+0.438  (0.000 ~ 1.409) | **0.014** |

# All relative abundance data for species that were significantly different are presented in this table (RA > 0.001 %). Relative abundance was analyzed by the Mann-Whitney *U* test (*p* < 0.05). Thick letters; OTUs with RA > 1%. Thick numbers; Mann-Whitney U test (*p* < 0.05).
